# Uncovering strain- and age-dependent innate immune responses to SARS-CoV-2 infection in air-liquid-interface cultured nasal epithelia

**DOI:** 10.1016/j.isci.2024.110009

**Published:** 2024-05-17

**Authors:** Jessie J.-Y. Chang, Samantha L. Grimley, Bang M. Tran, Georgia Deliyannis, Carolin Tumpach, An N.T. Nguyen, Eike Steinig, JianShu Zhang, Jan Schröder, Leon Caly, Julie McAuley, Sharon L. Wong, Shafagh A. Waters, Timothy P. Stinear, Miranda E. Pitt, Damian Purcell, Elizabeth Vincan, Lachlan J.M. Coin

**Affiliations:** 1 Department of Microbiology and Immunology, University of Melbourne at The Peter Doherty Institute for Infection and Immunity, Melbourne, VIC 3000, Australia; 2 Department of Infectious Diseases, University of Melbourne at The Peter Doherty Institute for Infection and Immunity, Melbourne, VIC 3000, Australia; 3 Victorian Infectious Diseases Reference Laboratory, Royal Melbourne Hospital at The Peter Doherty Institute for Infection and Immunity, Melbourne, VIC 3000, Australia; 4 Computational Sciences Initiative (CSI), The Peter Doherty Institute for Infection and Immunity, The University of Melbourne, Melbourne, VIC 3000, Australia; 5 Molecular and Integrative Cystic Fibrosis Research Centre, University of New South Wales, Sydney, NSW 2052, Australia; 6 School of Biomedical Sciences, Faculty of Medicine and Health, University of New South Wales, Sydney, NSW 2052, Australia; 7 Department of Respiratory Medicine, Sydney Children’s Hospital, Sydney, NSW 2031, Australia; 8 Australian Institute for Microbiology and Infection, University of Technology Sydney, Sydney, NSW 2007, Australia; 9 Curtin Medical School, Curtin University, Perth, WA 6102, Australia; 10 Department of Clinical Pathology, University of Melbourne, Melbourne, VIC 3000, Australia

**Keywords:** Immune response, Immunology, Transcriptomics, Virology

## Abstract

Continuous assessment of the impact of SARS-CoV-2 on the host at the cell-type level is crucial for understanding key mechanisms involved in host defense responses to viral infection. We investigated host response to ancestral-strain and Alpha-variant SARS-CoV-2 infections within air-liquid-interface human nasal epithelial cells from younger adults (26–32 Y) and older children (12–14 Y) using single-cell RNA-sequencing. Ciliated and secretory-ciliated cells formed the majority of highly infected cell-types, with the latter derived from ciliated lineages. Strong innate immune responses were observed across lowly infected and uninfected bystander cells and heightened in Alpha-infection. Alpha highly infected cells showed increased expression of protein-refolding genes compared with ancestral-strain-infected cells in children. Furthermore, oxidative phosphorylation-related genes were down-regulated in bystander cells versus infected and mock-control cells, underscoring the importance of these biological functions for viral replication. Overall, this study highlights the complexity of cell-type-, age- and viral strain-dependent host epithelial responses to SARS-CoV-2.

## Highlights

• SARS-CoV-2 elicits age-, strain- and cell-type-dependent host gene expression
• Ciliated and secretory-ciliated cells are highly infected in human nasal epithelia
• Alpha-variant upregulates host protein-refolding genes in children
• Oxidative phosphorylation-related genes may be mediators of host-viral competition

## Graphical abstract

**Figure:**
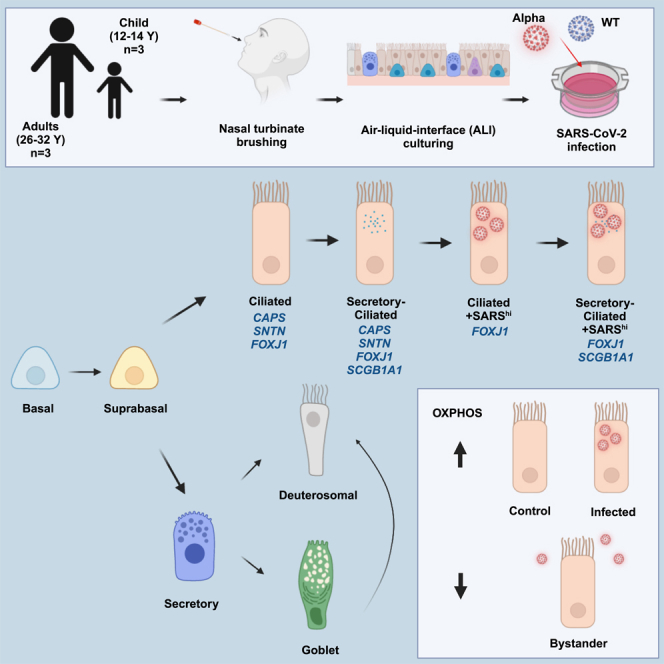

## Introduction

The single-stranded RNA virus, severe acute respiratory syndrome coronavirus 2 (SARS-CoV-2) is prone to mutations, contributing to the continuous emergence of new variants. Within the past few years of the Coronavirus Disease 2019 (COVID-19) pandemic, outbreaks of cases attributed to variants such as Alpha, Beta, Delta, and Omicron have led to ongoing waves of global upheaval. Although the dominant circulating variants are continuously changing over time, it is critical to gather information on earlier prevalent variants to help understand the dynamics of the emergence of future variants.^1^^,^^2^

The Alpha (B.1.1.7 lineage) is the first Variant of Concern (VOC), which was initially detected in the United Kingdom from a sample taken in September 2020 (VOC-202012/01).^3^ This variant has 23 genome mutations, including 14 non-synonymous mutations, 3 deletions, and 6 synonymous mutations.^4^^,^^5^ The N501Y mutation, located within the Receptor Binding Motif (RBM) of the Receptor Binding Domain (RBD) of the Spike (S) protein has been shown to dramatically increase binding affinity to the host cell receptor Angiotensin-Converting Enzyme 2 (ACE2) in humans as well as other species such as mice;^6^ thus the Alpha variant has a higher transmissibility and wider host range compared to pre-existing and co-circulating strains.^7^ Despite this increased transmission rate, there is no clear consensus regarding increased disease severity with Alpha-variant infections.^8^^,^^9^^,^^10^^,^^11^^,^^12^^,^^13^^,^^14^ Furthermore, relative to other VOCs, there is a lack of reports on whether the Alpha variant is implicated with differences in susceptibility, transmissibility, symptoms, infectiousness and host immune response when taking age into account (i.e., adults and children),^15^^,^^16^ which warrants further investigation into these effects to formulate a robust conclusion on strain- and age-effects.

There have been several studies applying single-cell RNA-sequencing (scRNA-seq) to examine host responses to SARS-CoV-2^17^^,^^18^^,^^19^^,^^20^^,^^21^ using infected cells collected directly from patients^20^^,^^22^ or from 3D organoids/air-liquid-interface (ALI)-cultures.^23^ While *in vivo* samples provide the biological relevance and complexity of host-pathogen interactions, ALI-culture settings allow a tightly controlled environment for assessing viral infections at the epithelial level without the interference of immune cells and allows the control of viral strain applied to the cells. Also, the time of infection/exposure to virus can also be accurately determined, providing an enhanced temporal assessment. Furthermore, ALI-cultures allow the growth of “mini human organs” derived from the same donors, which guarantee the genetic consistency between different treatments. Leveraging these benefits of *in vitro* cultures, visualizing combinations of different modalities, such as age and strain, can be simplified.

Previously, host responses to SARS-CoV-2 infections between children and adults have been compared using scRNA-seq.^20^^,^^22^ Child airway cells exhibited pre-primed immune responses, with stronger innate anti-viral/interferon (IFN) responses with SARS-CoV-2 infection than adults.^20^^,^^22^ However, these studies involve samples collected directly from infected donors with wide age spectrums, especially in children cohorts e.g., 4 weeks to 17 years,^20^ or up to 18 years.^22^^,^^24^ This can lead to high variation within groups, since younger children may exhibit different responses to older children.^25^ Further work is thus required for investigating age-dependent host responses to SARS-CoV-2 using scRNA-seq.^26^

Therefore, we sought to investigate the effects of two different SARS-CoV-2 strains – the ancestral strain first isolated in Australia (VIC01, referred to from here on as WT)^27^ and the Alpha variant (VIC17991) - during the infection of ALI-cultured primary nasal epithelial cells. The cells were derived from donors with narrow age brackets, including younger adults (26–32 Y) and older children (12–14 Y). Here, we report the strain- and cell-type-dependent epithelial host response to SARS-CoV-2 in children and adults.

## Results

### Severe acute respiratory syndrome coronavirus strains show varied infection levels in adult and child nasal epithelia

**Figure 1:**
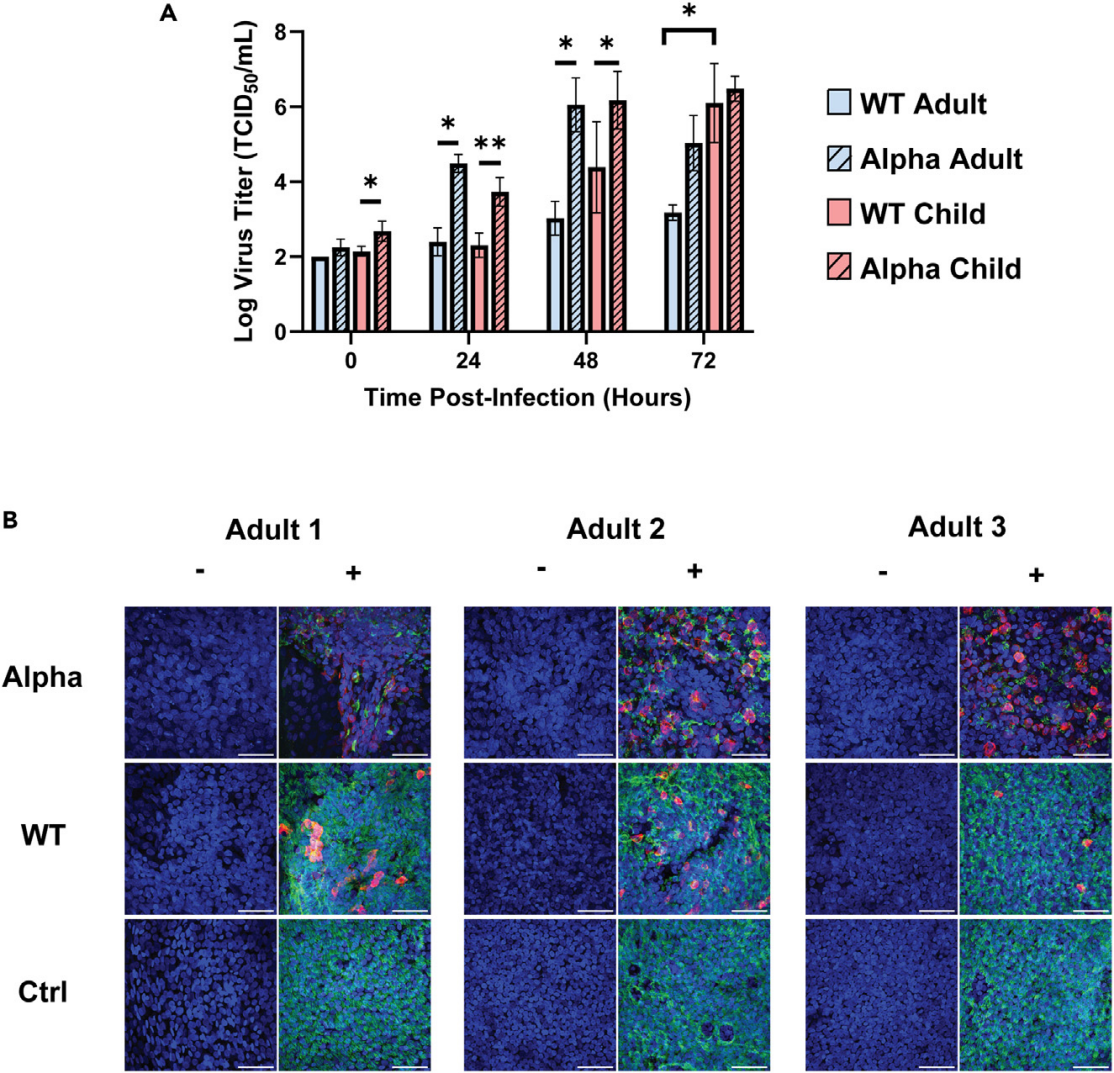
Higher viral titers correlate with the reduction of epithelial cilia in Alpha-vs. WT strain-infected ALI-cultures in adults (A) TCID_50_ results from apical washes at 0, 24, 48, 72 hpi comparing adults and children with WT and Alpha infections. Data are represented as mean log virus titer ± standard deviation (SD), *n* = 3, where each n represents each donor within an age-group (adult/child) averaged from 1 to 3 technical replicates. Statistical testing was carried out with the two-tailed paired T-test for WT vs. Alpha analyses within each age-group and Welch’s T-test for adult vs. child analyses (p = ∗ ≤0.05, ∗∗ ≤0.01). Only significant values are notated (*p* ≤ 0.05). See also Figure S1. (B) Immunofluorescent confocal microscopy staining at 40× magnification of ALI-HNECs of Alpha variant-infected cultures compared with WT strain in adults at 72 hpi. Mock-control cells were harvested at 7 days. Stained for α-tubulin (AcTub, green), nucleoprotein (NP, red) and nuclei (DAPI, blue). Negative controls are indicated by “-"’ and complete stains are indicated with “+”. Scale bar: 50 μm. Images are repeated in Figures S2B–S2G as individual channels and final combined channel images for clarity. See also Figure S2.

The nasal epithelial ALI-cultures derived from three healthy adults aged from 26 to 32 years and three children with ages ranging from 12 to 14 years were infected on the apical side with either Alpha or WT SARS-CoV-2 strains. The levels of infectious viral titer across a time-course were determined by TCID_50_ of apical washes (STAR Methods). We found that WT and Alpha strains expressed contrasting infection efficiency in adults and children, especially at 24–48 hpi (*p* ≤ 0.05, Figures 1A and S1A–S1D). Of note, while WT was unable to replicate efficiently in adults at 72 hpi, it showed high levels of infection in children at the same timepoint (*p* ≤ 0.05, Figures 1A and S1A–S1D). On the other hand, the cultures infected with the Alpha variant similarly exhibited high infection levels in both adults and children (*p* > 0.05, Figures 1A and S1A–S1D).

We then used confocal microscopy to visualize the effect of viral infection on the hosts after 72 hpi (Figures 1B and S2A–S2G). Cilia disruption is a key presentation of SARS-CoV-2 infection, associated with antagonizing mucociliary clearance.^28^ We noted that the Alpha-infected cells showed rapid loss of cilia compared with WT in adults (Figure 1B). The rate of depletion suggested to be faster in adults than children despite similar titer values with the Alpha variant (Figure 1A), as we observed the diminished level of cilia disruption in child cells in contrast to adults (Figures S2A and S2E–S2G). Our phenotypic results indicate the different impacts of the Alpha-variant on nasal epithelial cells in adults and children under controlled, *in vitro* environments.

### Highly infected sub-lineages of ciliated cells expand in response to infection

**Figure 2:**
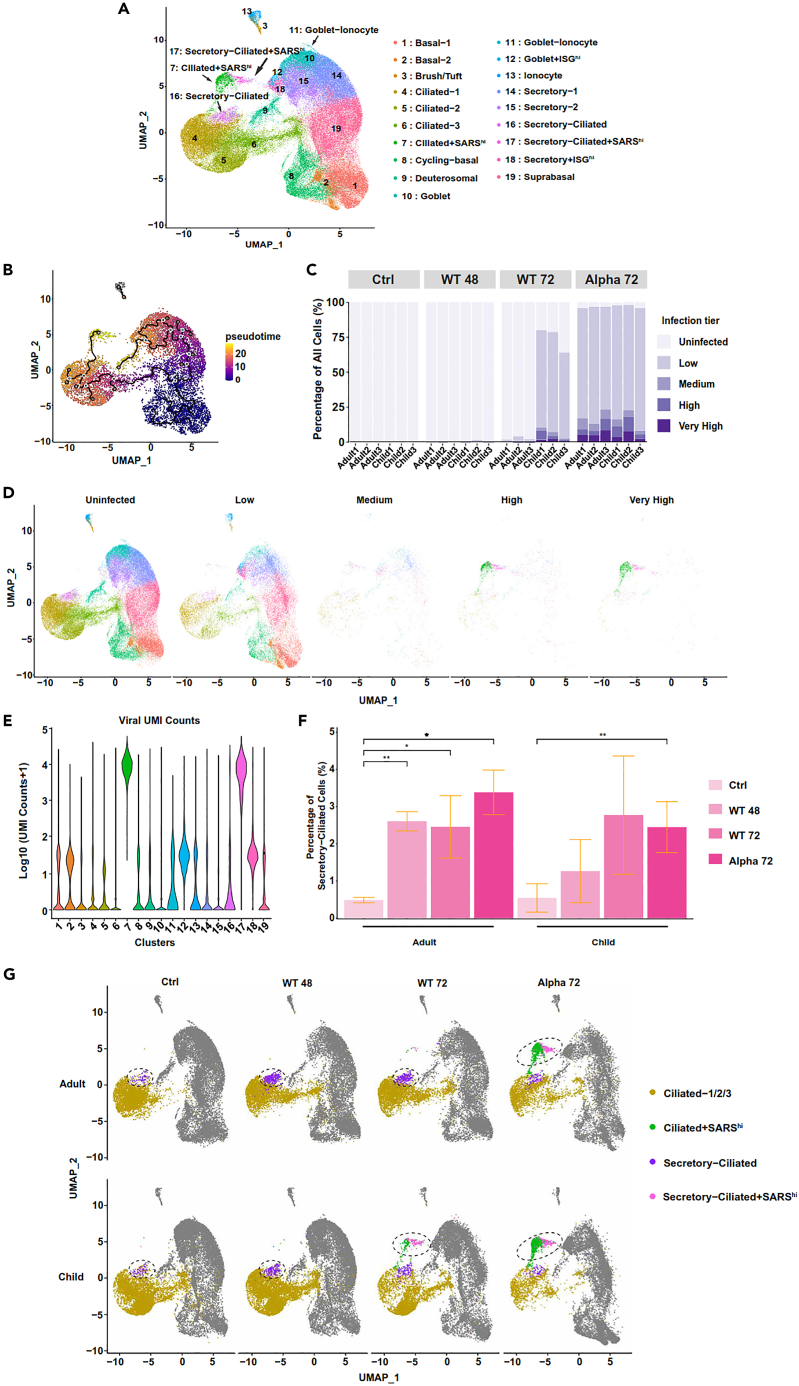
Infection levels per cell-type and condition in the human nasal epithelium (A) Uniform Manifold Approximation and Projection (UMAP) of cells from all samples after unsupervised clustering with known cell markers. See also Data S1 and S2, Figure S4 & Tables S1 and S2. (B) Pseudo-time trajectory of down-sampled subset of cells, showing the bifurcation of differentiation progression into ciliated and secretory arms. Black circles indicate branch nodes, and gray circles indicate the different outcomes of the trajectory. The numbers within the circles are only for reference purposes. See also Figure S5. (C) Percentage of infected cells in adults and children (*n* = 3) in each condition per donor, stratified by infection tier. See also Data S3 & Table S2. (D) UMAP of cells split by infection tier after filtering (STAR Methods) - uninfected (<10 viral counts per cell), low (<100 viral counts per cell), medium (<1000 viral counts per cell), high (<10,000 viral counts per cell), and very high infection levels (≥10,000 viral counts per cell). See also Table S2. (E) Log_10_ (UMI counts +1) of viral reads, with legends shown as in Figure 2A. See also Table S2. (F) Percentage of all secretory-ciliated cells between each treatment condition in adults and children. Data are represented as mean ± SD, *n* = 3, where each n represents each donor within an age-group (adult/child), two-tailed paired T-test (p = ∗ ≤0.05, ∗∗ ≤0.01). See also Figures S3 and S4 & Data S4. (G) UMAP plots revealing the differentiation pathway for forming high viral load cells in the ciliated arm of the pseudo-time trajectory bifurcation per treatment condition. Dotted circles represent the most dominant downstream cell cluster(s) along the pseudo-time trajectory. See also Figure S3.

We used scRNA-seq to uncover the impact of infection in different cell-types within the human nasal epithelia. The traditional landscape of the human nasal epithelia is mainly composed of basal, ciliated, and secretory cells.^29^ Additionally, rarer cell-types such as ionocytes,^17^^,^^30^ deuterosomal cells^31^^,^^32^ and transitional cell-types with cell signatures from more than one cell-type may be present.^28^ We first clustered and assigned cell-types to our datasets by *Seurat* (Data S1),^33^ based on cell markers from public scRNA-seq datasets and within the literature.^17^^,^^31^^,^^32^ We were able to capture all common cell-types, such as ciliated, basal and secretory cells (Figure 2A & Data S1; Tables S1 and S2),^31^^,^^32^ as well as the transitional cell-types, including secretory-ciliated cells^28^ and goblet-ionocyte cells, which have relatively small population sizes in the human nasal epithelium (Figure 2A & Data S2).

Then, to understand the progression of differentiation in our ALI-cultures, we performed a trajectory analysis using *Monocle3*.^34^ The pseudo-time trajectory revealed a bifurcation of the trajectory originating from basal cells, where ciliated and secretory cell-types showed two distinct lineages with a lack of cross-over, in both mock-control and infected samples (Figure 2B). The progression of the ciliated arm of the bifurcation is seen to be basal → suprabasal → ciliated → secretory-ciliated. Interestingly, these results suggested that secretory-ciliated cells were derived from ciliated cells (Figure 2B). This runs contrary to the paradigm that secretory cells are precursors to ciliated cells,^35^ and instead supports the idea that secretory properties were acquired by ciliated cells, rather than stemming from secretory lineages.

Next, we determined the global levels of infection. We classified cells into four infection levels based on the number of Unique Molecular Identifiers (UMI) including uninfected, low, medium, high, or very high level of infection respectively (STAR Methods). At 48 hpi, we recovered minimal viral UMI counts in both adult and child samples infected with the WT strain (Figure 2C), much like the observation from viral titers (Figure 1A). For WT, the infection dramatically increased at 72 hpi, but the escalation of infection only occurred in the child ALI-cultures (Figure 2C & Data S3). On the other hand, for cultures infected with the Alpha variant, most cells were found to be infected, indicating the high infectivity of this variant within both children and adults (Figure 2C). These results are consistent with our phenotypic observations based on viral titers (Figure 1A), providing further validation of the distinctive infection of SARS-CoV-2 variants in children and adults.

We then identified the specific cell-types prone to SARS-CoV-2 infection. In both adult and child ALI-cultures, most cell-types exhibited low levels of infection (<100 viral UMI counts) (Figures 2C–2E; Table S2). However, two sub-clusters of ciliated (denoted as “Ciliated+SARS^hi^”) and secretory-ciliated (denoted as “Secretory-Ciliated+SARS^hi^”) cells showed significantly higher levels of infection (Figures 2D and 2E). Although the susceptibility of ciliated cells and secretory-ciliated cells to SARS-CoV-2 has been noted previously in human airway epithelial cells (HAECs),^22^^,^^28^ our data shows the exceptionally high infection levels in these cell-types (Figure 2E). In particular, upon infection with SARS-CoV-2, we observed a statistically significant increase in the proportion of secretory-ciliated (Secretory-Ciliated & Secretory-Ciliated+SARS^hi^) cells, which was more evident in Alpha than WT infection (Figures 2F and S3, Data S2 and S4, *p* < 0.05). Although the magnitude of change was small (<4% of all cells), this suggests an enhanced generation/turn-over of secretory-ciliated cells due to infection with the Alpha variant. Interestingly, despite similar viral titers compared with the Alpha variant-infected cultures (Figure 1A), the significant expansion of secretory-ciliated cells was not observed in WT datasets at 72 hpi in children (Figure 2F). Furthermore, in relation to these results, Secretory-Ciliated cells (excluding Secretory-Ciliated+SARS^hi^ cells) increased in proportion with infection compared with the mock-control dataset in adults, but this observation was absent in children (Data S4 & Figure S3), suggesting that turn-over rate in these cells is faster in adults. Additionally, we observed the increase in Ciliated+SARS^hi^, Secretory-Ciliated+SARS^hi^, Secretory+ISG^hi^, Goblet+ISG^hi^ cells in infected cells at 72 hpi in both age-groups (moderated T-test, FDR <0.05, Data S2 and S4), which was again more pronounced in Alpha 72 hpi datasets compared with WT 72 hpi.

Within the pseudo-time trajectory, we observed the highly infected Ciliated+SARS^hi^ cluster downstream of Secretory-Ciliated cells, with apparent loss of secretory marker expression, followed by a highly infected Secretory-Ciliated+SARS^hi^ cluster (Figures 2B and S4). This trajectory suggests the secretory markers of this cell-type may be transiently gained and lost, which is more evident with infection. Hence, upon SARS-CoV-2 infection, we describe the general progression of the ciliated arm as; basal → suprabasal → ciliated → secretory-ciliated → highly infected ciliated → highly infected secretory-ciliated cells (Figures 2B and 2G). In contrast, the infection levels of the basal → suprabasal → secretory → goblet → deuterosomal pseudo-time branch is generally lower than the aforementioned ciliated branch, although a small proportion of cells of Secretory+ISG^hi^ (4.6%), Goblet+ISG^hi^ (3.4%), Deuterosomal (2.3%), Goblet-Ionocyte (1.3%) clusters revealed high-very high levels of infection (Table S2). The susceptibility of secretory/goblet cells in addition to ciliated and transitional cell-types is consistent with previous studies.^36^ Conversely, the suprabasal cells were the largest subset of cells (>20,000), but remained largely uninfected by both WT and Alpha variants (Figure 2E; Table S2).

To further investigate the trajectory of secretory-ciliated cells, all cells within secretory, ciliated, secretory-ciliated, goblet cell populations were isolated and RNA velocity analysis was performed using *scVelo*.^37^ Velocity analysis revealed mainly strong temporal dynamics within cell-types, with little transition across cell-type boundaries, apart from the ciliated → secretory-ciliated directionality (Figure S5A). Partition-based graph abstraction (PAGA) analysis indicated a convergence of directionality from ciliated and secretory cells to secretory-ciliated cells (Figure S5B). These results partially support the ciliated → secretory-ciliated pathway shown in the *Monocle* trajectory analysis (Figure 2B). Whilst less evident in the trajectory analysis, the secretory → secretory-ciliated directionality was clearly shown in the PAGA analysis, in line with the typical differentiation pathway in airway epithelial cells, where secretory cells are believed to be precursors of ciliated cells.^32^ At the gene level, the transcriptional dynamics of ciliated cell marker *SNTN* showed a direction of secretory → secretory-ciliated → ciliated, and the secretory cell marker *SCGB1A1* revealed the opposite direction of secretory-ciliated → secretory, as expected (Figure S5C). Furthermore, we observed a higher proportion of unspliced transcripts (∼42–47%) in the “SARS^hi^” clusters compared with all other clusters, which again aligned with the expectation of infection-induced acceleration of transcription (Figure S5D).

### Innate immune response differences between infected vs. bystander cells

**Figure 3:**
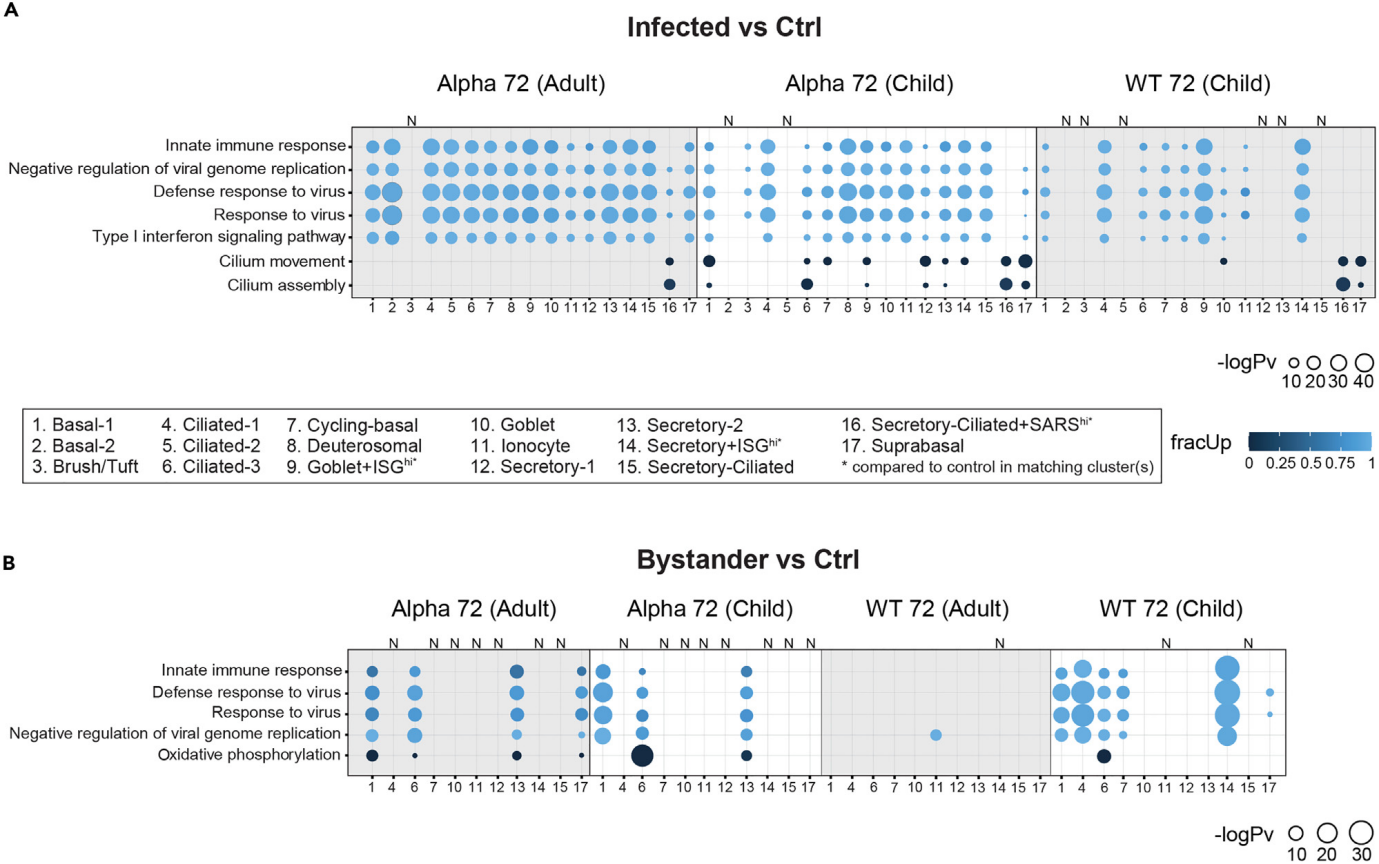
Significantly enriched GO biological terms analyzed via *multiGO* using significant DE results in infected and bystander vs. mock-control cells (A) Infected vs. mock-control cells - strong enrichment of innate immune responses was observed in infected vs. mock-control cells, where DE genes were mainly up-regulated. Cilia-related genes were down-regulated in Secretory-Ciliated+SARS^hi^ cells. (B) Bystander vs. mock-control cells – similarly to infected vs. mock-control cells, strong enrichment of innate immune responses was observed in bystander vs. mock-control cells, where DE genes were mainly up-regulated. Oxidative phosphorylation-related genes were down-regulated. However, WT 72 hpi (adult) datasets lacked these responses. Bubble size indicates -log_10_ enrichment *p*-values, and the color of the bubble indicates the proportion of up-regulated genes in the term (i.e., fracUp). X axis shows the corresponding cell clusters matching in the legend and corresponds for both Figures 3A and 3B. Y axis shows the enriched GO terms. Thresholds of p_adj_ <0.05, enrichment *p*-value <0.005 and |logFC| >1 were used. Columns with no matching DE data available are denoted with “N”. High viral load and ISG populations (Goblet+ISG^hi^, Secretory+ISG^hi^, Secretory-Ciliated+SARS^hi^) were compared with mock-control cells from other related cell clusters (STAR Methods). A subset of the results from the top 35 terms are shown. A full list of enriched GO terms is available via the links in Table S3. See also Figures S6–S8.

We then proceeded to investigate the effect of SARS-CoV-2 on the host epithelial innate immune response at the transcript level. Firstly, we implemented gene ontology (GO) enrichment and reactome pathway analyses on infected vs. mock-control cells in both adult and child donors on a cell-type basis at 72 hpi to uncover the expression pattern. The gene sets used as input for the GO search were the significantly differentially expressed genes against a background of all the detectable genes in the RNA-seq data. Globally, most infected cell-types showed strong expression of genes involved in innate immune response-related GO terms, including *defense response to virus, response to virus, type I interferon signaling pathway*, *innate immune response* and *negative regulation of viral genome replication* (Figures 3A and S6A), with the exception of the high viral load Secretory-Ciliated+SARS^hi^ clusters across all datasets. These results suggested that the innate immune response is stronger in lowly infected epithelial cells, compared with highly infected cells.

In contrast, genes involved in *cilium movement* and *cilium assembly* were strongly down-regulated in Secretory-Ciliated+SARS^hi^ clusters. This observation was less evident in other cell-types in adult cultures infected with the Alpha variant, despite similar titers in child cultures (Figure 1A). Similarly, this response was diminished in child cultures infected with WT, shown by the absence of the enrichment of these GO terms (Figure 3A). As motile cilia has been found to be important for efficient viral replication,^38^ the down-regulation of related genes appeared to be part of the host defense response to SARS-CoV-2.

In the context of infections at the epithelial level, bystander cells are cells which remain uninfected in samples challenged with the pathogen (in this case SARS-CoV-2).^17^ These cells are important for investigating the host-response to SARS-CoV-2 without the interference of viral-replication/transcription activity. To understand whether bystander cells are impacted by exposure to SARS-CoV-2, we then compared the gene expression of bystander cells vs. mock-control cells. Bystander cells revealed similar responses to infected cells (Figure 3A), with most cell-types exhibiting the up-regulation of genes related to innate immune responses (Figure 3B). Similarly, reactome pathways such as *interferon alpha/beta signaling* and *interferon signaling* were enriched in these datasets in the same cell clusters (Figure S6B). Consistent with existing evidence that bystander cells are affected by the paracrine activity of cytokines which are released from infected neighboring cells,^39^ these results suggested that the exposure but not the infection of SARS-CoV-2 can still elicit an up-regulation in anti-viral gene expression (Figures S7 and S8). We note that the WT 72 hpi dataset in adults (which showed low infection (Figure 1A)) had a diminished response with only Ionocytes exhibiting the enrichment of the *negative regulation of viral genome replication* compared with other datasets with strong infection. This suggests that initial exposure or low infection are insufficient for eliciting robust innate immune responses within bystander cells. Furthermore, in strongly infected cultures, the down-regulation of genes involved in *oxidative phosphorylation* was observed. This suggests the reduced oxidative phosphorylation activity in bystander cells compared with mock-control cells, which did not appear to be strongly enriched in infected vs. mock-control comparisons (Figure 3A; Table S3).

We carried out further investigation of cell-type-specific expression of cytokine-related genes by infection level (bystander, low, high/very high) in the adult Alpha and WT 72 hpi datasets (Figures S7 and S8). Certain cytokine-related genes were expressed across multiple cell-types in all three infection levels, including *B2M* (beta-2 microglobulin), *BST2* (bone marrow stromal cell antigen 2), *RTN4* (reticulon-4), and *HLA-A* (major histocompatibility complex, class I, A) (Figures S7 and S8). However, as expected, the infected groups showed broader cell-type range and cytokine-related gene expression compared with bystander cells (as also shown in Figures 3A and 3B). We noted differences between bystander and infected groups, such as *HLA-E* (major histocompatibility complex, class I, E) and *HLA-F* (major histocompatibility complex, class I, F) being expressed highly in infected groups, but less so in the bystander group in the Alpha 72 hpi dataset (Figure S7). Interestingly, there was little variation in the expression of most genes explored between low and highly/very highly infected cells in the two infected groups in Alpha 72 hpi, whereas a starker contrast was observed in WT 72 hpi datasets (Figures S7 and S8). However, in highly/very-highly infected secretory-ciliated cells, we observed a higher expression of *MAVS* (Mitochondrial antiviral-signaling protein) in Alpha 72 hpi datasets. Furthermore, Ionocytes in particular showed higher expression of *MIF* (macrophage migration inhibitory factor) and *CLNK* (Cytokine Dependent Hematopoietic Cell Linker) than other cell-types, but with similar expression levels between different infection groups, highlighting a cell-type-dependent effect.

**Figure 4:**
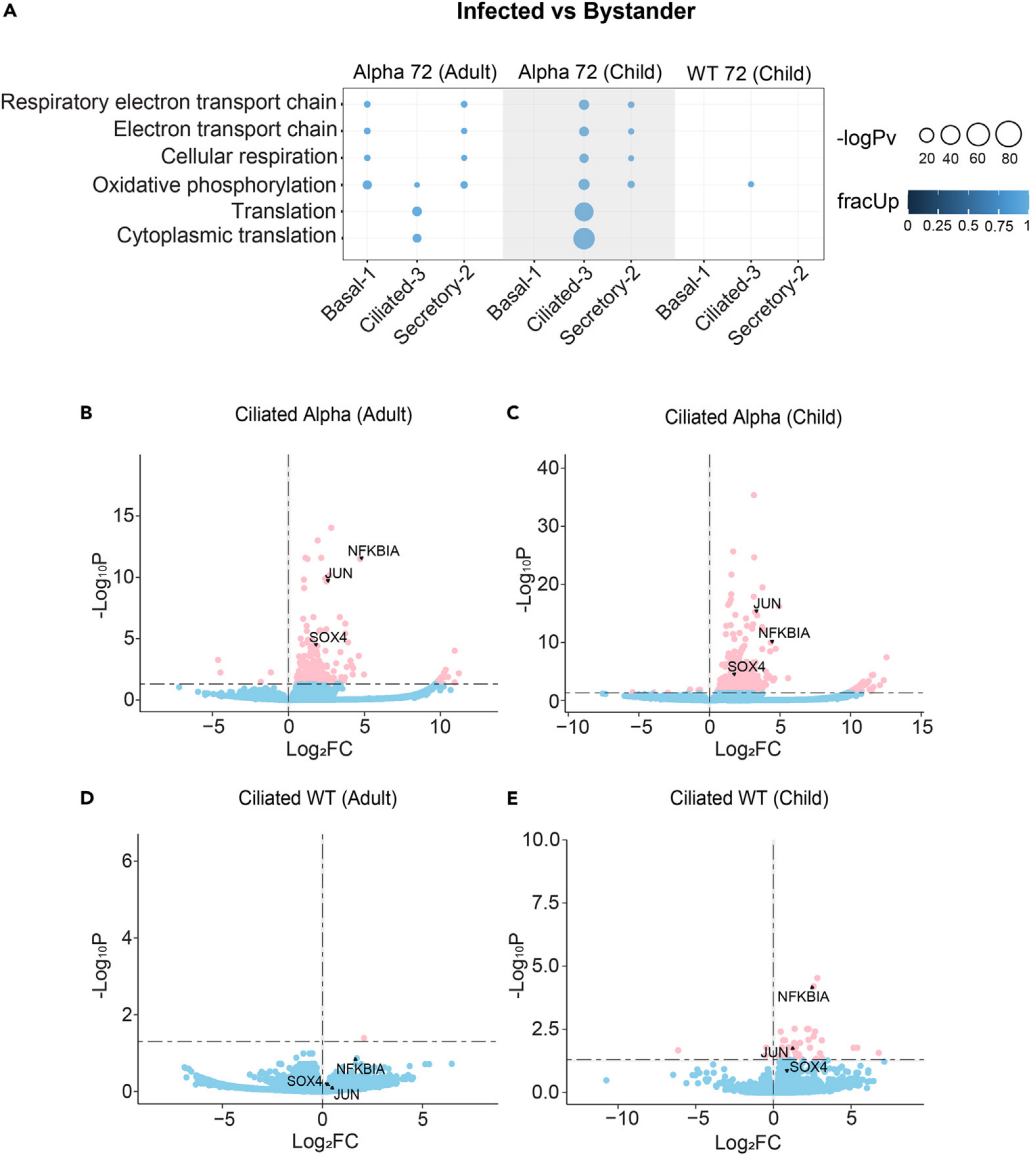
Up-regulation of genes in infected vs. bystander cells (A) Enriched GO terms in Alpha- and WT-infected cells in adults and children vs. respective bystander cells. Bubble size indicates -log10 enrichment *p*-values, and the color of the bubble indicates the proportion of up-regulated genes in the term (i.e., fracUp). Thresholds of pv_thresh = 0.05, enrichment pv_thresh = 0.005 and logFC_thresh = 1 were used. A subset of the results from the top 35 terms are shown. A full list of enriched GO terms is available via the link in Table S3. (B–D) Volcano plots of significantly differentially expressed genes in ciliated cells at 72 hpi within (B) Alpha-infected cells in adults, (C) Alpha-infected cells in children, (D) WT-infected cells in adults, and (E) WT-infected cells in children. Up-regulation of *NFKBIA, JUN* and *SOX4* in Alpha-infected data and *NFKBIA* and *JUN* in WT-infected child data were observed. Thresholds of p_adj_ <0.05 and logFC = 0 were used. X axis shows the log_2_FC in infected (Alpha/WT) vs. bystander datasets, and Y axis shows the -log_10_padj from the DE analysis. Blue dots indicate genes which meet only the log_2_FC threshold and pink dots indicate the genes which meet both the p_adj_ and log_2_FC thresholds. See also Figure S6C.

To confirm the contribution of these pathways in infected cells, we then compared infected and bystander cells directly. We found that genes involved in oxidative phosphorylation were up-regulated, particularly in Ciliated-3 cells across ages and strains (Figure 4A). Similarly, the reactome pathway analysis showed the enrichment of pathways such as *the citric acid (TCA) cycle and respiratory electron transport* and *respiratory electron transport, adenosine triphosphate (ATP) synthesis by chemiosmotic coupling, and heat production by uncoupling proteins.* Furthermore, infection-related (*infectious disease* and *interleukin-1 signaling*) and translation-related (*viral mRNA translation* and *translation*) pathways were enriched, with the genes involved being also largely up-regulated in both age-groups (Figure S6C). Particularly, *NFKBIA* (NFKB inhibitor alpha)*, JUN* (Jun proto-oncogene, AP-1 transcription factor subunit), and *SOX4* (SRY-box transcription factor) were found to be among some of the most strongly up-regulated genes in infected ciliated cells, especially in Alpha-infected cells (Figures 4B and 4C). The expression is similar to the study by Ravindra et al.^17^ which examined WT SARS-CoV-2-infected ALI-human bronchial epithelial cells (HBECs), despite the differences in the location of the host cells within the human airway (HBEC instead of human nasal epithelial cells (HNEC)). However, in our study, we noted that only *NFKBIA* and *JUN* showed increased expression in child infected cells with WT infection, with no significant differential expression (DE) found in adult cells (Figures 4D and 4E). These differences may be attributed to the tissue type (nasal vs. bronchial cells), method for DE (pseudo-bulk vs. non-pseudo-bulk) or strain-differences (ancestral Australian vs. ancestral USA).

### Alpha variant induces increased protein folding and innate immune responses

**Figure 5:**
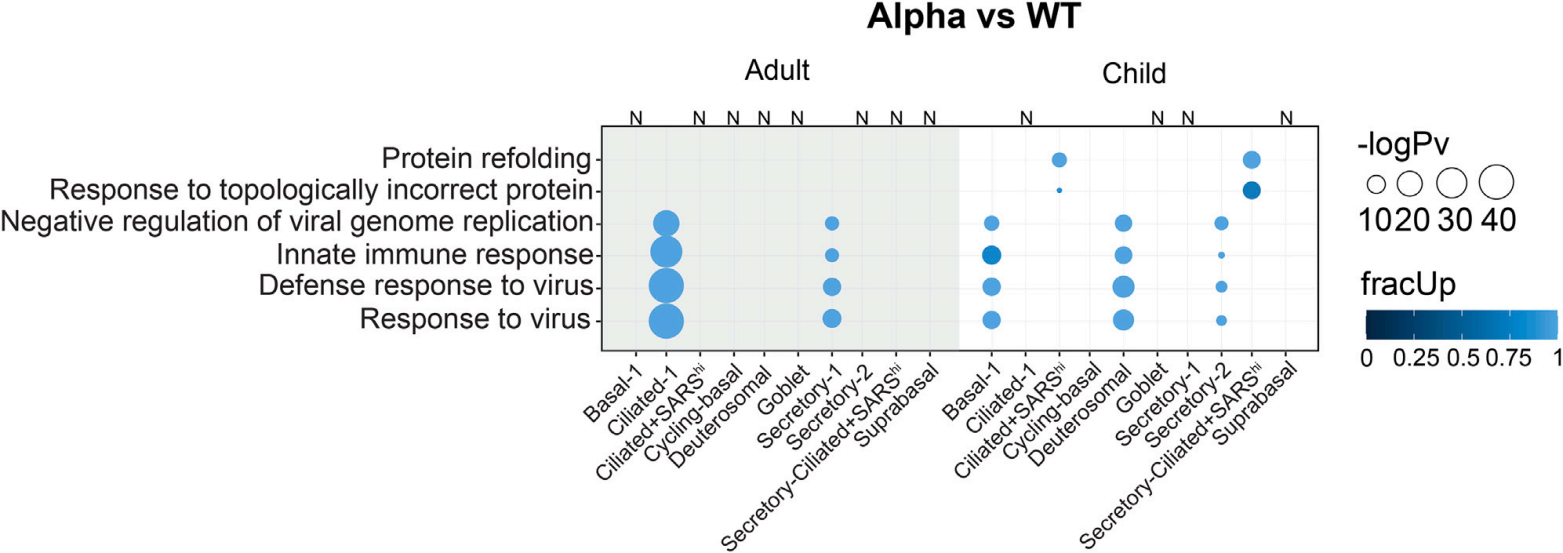
Significantly enriched GO biological terms analyzed via *multiGO* using significant DE results between Alpha-vs. WT-infected cells Up-regulation of genes involved in innate immune response observed in Alpha-infected cells. Protein refolding-related genes were also up-regulated in child cultures within highly infected cell clusters (Ciliated+SARS^hi^ & Secretory-Ciliated+SARS^hi^). Bubble size indicates -log_10_ enrichment *p*-values, and the color of the bubble indicates the proportion of up-regulated genes in the term (i.e., fracUp). Thresholds of p_adj_ < 0.05, enrichment *p*-value <0.005, |logFC| >1 were used. Columns with no matching DE data available are denoted with “N”. A subset of the results from the top 35 terms are shown. A full list of enriched GO terms is available via the link in Table S3. See also Figure S9.

Finally, we uncovered the differences in host responses to the Alpha variant and WT-strain of SARS-CoV-2. Anti-viral terms such as *innate immune response and defense response to virus* were enriched in both adults (Ciliated-1, Secretory-1) and children (Basal-1, Deuterosomal, Secretory-2) (Figure 5). In terms of reactome pathways*, interferon signaling, interferon alpha/beta signaling, and antiviral mechanism by IFN-stimulated genes* were enriched in the same cells (Figure S9). The expression levels were higher in the Alpha variant infections compared with the WT strain. These results highlight the heightened involvement of anti-viral host responses to Alpha infections. Additionally, we found that genes involved in *protein refolding* and *response to topologically incorrect protein* were largely up-regulated in Ciliated+SARS^hi^ and Secretory-Ciliated+SARS^hi^ datasets involving children (Figure 5). This suggests that the Alpha variant elicits a greater post-translational activity related to refolding aberrantly folded/unfolded proteins in the most infected clusters of cells, at least within children. We note that we did not have enough WT-infected cells in those clusters in adult cultures to compare with that of the children.

## Discussion

Our work uncovers the dynamics of cellular differentiation within ALI-HNEC cultures upon *in vitro* SARS-CoV-2 infection, and the interplay between viral load and host epithelial innate immune responses at cell-type, age, and strain levels. We demonstrated the strain- and age-dependent responses to SARS-CoV-2 within heterogeneous populations of HNECs. Cell-type distributions were similar between the two age-groups, where ciliated and secretory-ciliated clusters with high levels of viral infection were clearly observed in our data. Alpha-variant infections showed higher viral titers/loads compared with WT in particularly adult cultures, with minute differences within child cultures. Interestingly, the Alpha-variant appeared to induce a protein refolding response in the host in comparison with WT in children. Finally, via analyzing the responses in infected vs. bystander cells, oxidative phosphorylation appeared to be a key pathway in which the host and viral-responses intersect during SARS-CoV-2 infections.

We observed strain-dependent host responses to SARS-CoV-2 infection. Both the viral titers (Figure 1A) and RNA-seq data (Figure 2C) showed that the Alpha-variant infection resulted in generally higher viral loads than WT in both adult (26–32 Y) and child (12–14 Y) ALI-cultures, with the difference observed earlier in viral titers (24 & 48 hpi) than RNA-seq data (72 hpi). This difference was amplified in adults compared with children (Figure 2C). The elevated viral titers with Alpha-infections compared with WT were expected, due to the increased transmissibility in the variant via enhanced receptor binding affinity due to the N501Y mutation.^7^^,^^15^ Furthermore, viral loads have shown to be higher in Alpha-variant infections than non-VOC strains in other studies,^40^^,^^41^^,^^42^^,^^43^^,^^44^ which appeared to be more evident in adults compared with children,^43^ supporting our observations.

We also noted age-dependent responses to WT infection at 72 hpi, where our data revealed generally higher viral titers/loads in children than adults (Figures 1A and 2C). However, contrary to our WT results, Zhu et al. (2022)^45^ revealed that the Queensland (Australia) ancestral strain resulted in lower viral titers in child (5.6 ± 2.7 Y) vs. adult (37.9 ± 16.5 Y) ALI-HNECs. On the other hand, Capraro et al. (2021)^46^ showed lower viral titers in older adult (58.14 ± 3.30 Y) ALI-HNECs compared with children (5.40 ± 3.97 Y) and young adults (31.89 ± 4.14 Y), showing an age-dependent decline of viral titer.^46^ The disparities between studies may be influenced by various factors including – the multiplicity of infection (MOI), viral strain/stock, the different age brackets used for child donors and donor-dependent behavior in ALI-culture studies.^47^ Therefore, with these points in mind, further work will be required to fully elucidate the age-dependent effects of SARS-CoV-2 infection.

In child ALI-cultures, the Alpha-variant infected cells showed the up-regulation of genes involved in *protein refolding* and *response to topologically incorrect protein*, compared with WT-infected cells, in the clusters with the highest level of infection (i.e., Ciliated+SARS^hi^ and Secretory-Ciliated+SARS^hi^) (Figure 5). Under normal conditions, the protein refolding response is inactivated, and is switched on after an accumulation of unfolded/misfolded proteins occurs under endoplasmic reticulum (ER) stress.^48^ The aggregation of unfolded proteins may occur as a host defense mechanism, or viral manipulation to increase replication or viral immune evasion.^49^^,^^50^ The induction of the unfolded protein response (UPR) due to ER stress has been documented with the spike protein of SARS-CoV-1^51^ and also SARS-CoV-2.^52^ Overall, this increased activity in child HNECs may be in part due to the increased transmissibility of the Alpha strain increasing the build-up of misfolded/unfolded proteins. This may be also due to the accumulation of excess viral proteins during infection, overwhelming the cellular architecture, and therefore negatively affecting both host and viral post-translational modifications and appropriate protein folding. We note that the matching data with Ciliated+SARS^hi^ and Secretory-Ciliated+SARS^hi^ clusters in adults was unavailable. While we cannot comment on an age-dependent/independent protein-refolding activity, we speculate these processes could have been reciprocated in the adult datasets had we been able to analyze these datasets, due to strain-dependent viral-load being observed between WT and Alpha-infections in adults (Figure 2C).

The presence of highly infected cell clusters of ciliated and secretory-ciliated attributes - Ciliated+SARS^hi^ and Secretory-Ciliated+SARS^hi^ – were in line with previous reports of ciliated cells being most prone to SARS-CoV-2 infection (Figure 2A).^19^^,^^38^^,^^53^ While the literature mainly focuses on the infection of ciliated cells, we note recent reports of the SARS-CoV-2 infection of secretory-ciliated cells in the human airway epithelia. These cells have been characterized loosely as “transient secretory cells,” expressing markers of ciliated (*FOXJ1, TUBA1A/TUBB4B*), goblet (*MUC5AC*) and club/goblet (*SCGB1A1*) cells.^30^ The trajectory analysis showed an unexpected differentiation pathway of ciliated → secretory-ciliated cells, described by Robinot et al. (2021),^28^ instead of secretory → secretory-ciliated cells, as described by Lukassen et al. (2020),^30^ and Thaler et al. (2023) (Figure 2B).^54^ The RNA velocity analysis provided evidence of both directionalities (Figures S5A and S5B), with stronger evidence of ciliated → secretory-ciliated cells (Figure S5A). Therefore, further efforts are still required to completely understand the lineage of this transitional cell-type. Although forming a small percentage of overall cells (<4%), the proportion of secretory-ciliated cell populations (Secretory-Ciliated+SARS^hi^ & Secretory-Ciliated) significantly increased with Alpha infections (Figure 2F). This indicated another strain-dependent effect as we observed similar viral loads between Alpha and WT infections in child cultures at 72 hpi (Figure 2C), and yet we were unable to find a significant enrichment of these cells in the WT 72 hpi dataset (Figure 2F). One speculation for the expansion of secretory-ciliated cells upon infection may be that the ciliated cells may acquire more secretory properties to facilitate the increase of mucus production which is favorable for trapping viral particles during infection. Otherwise, it has been recently reported that mucociliary activity supports SARS-CoV-2 spread in ALI-cultured human airway epithelia,^38^ where motile cilia facilitates the binding of SARS-CoV-2 for entry and infection. The microvilli and mucus dispersion enhance viral spread, supporting the high viral loads in secretory-ciliated cells. Although our groups of secretory-ciliated cells express the general marker for secretory cells (*SCGB1A1*) more than goblet-related markers (*MUC5AC*, *MUC5B*) (Figure S4), the fact that secretory cells can be precursors for goblet cells may provide a potential reason for this phenomenon.^32^ We speculate that had we sampled more cells or had a subsequent time-point beyond 72 hpi, we may have observed an increase in *MUC5AC*-*FOXJ1* goblet-ciliated cells.

In SARS-CoV-2 infection, cytokines are produced as a response to the recognition of viral pathogen-associated molecular patterns (PAMPs) and are signaling molecules for bridging the innate and adaptive immune systems.^55^ Our results along with other studies^17^^,^^56^ revealed the paracrine activity of ISGs in bystander cells. However, some key cytokine-related genes such as non-canonical major histocompatibility complex (MHC) class I molecules – *HLA-E* and *HLA-F* were expressed highly in infected cells but less evidently in Alpha-variant-exposed bystander cells, while the canonical MHC class I molecule gene *HLA-A* was expressed more widely in both bystander cells as well as infected cells (Figure S7). Considering that non-canonical HLA molecules pose a more inhibitory effect on immune cells (e.g., natural killer (NK) cells) through interaction with inhibitory receptors than their canonical counterparts,^57^ this co-expression of canonical and non-canonical HLA molecules can be thought to provide the cells with protection from NK-mediated cytotoxicity, while maintaining CD8^+^ T cell-specific killing (as discussed by Wyatt et al.).^57^ Furthermore, Ionocytes showed comparatively higher expression of *MIF* and *CLNK* compared with other cell-types, consistently across different infection levels (Figures S7 and S8), in which these genes are largely associated with immune cells. While this has been noted in this cell-type, unrelated to infection,^58^ the stable expression of these genes across infection levels suggests a purpose of these cells apart from their more commonly known function – i.e., ion transmembrane transporter activity.^59^ One potential reason for this may be due to a pre-priming state, as within airways of children compared with adults, to prepare for a rapid recruitment of immune cells upon infection. Although this cell-type was largely uninfected, with ∼30% of infected cells overall (Table S2), these results warrant further investigation into the immune-related roles of Ionocytes.

We also demonstrated the importance of oxidative phosphorylation during the SARS-CoV-2 infection. Oxidative phosphorylation is an integral part of the energy production process.^60^ This process produces ATP and reactive oxygen species (ROS), where an excessive level of the latter can lead to oxidative stress and inflammation.^61^ Furthermore, ROS has been shown to suppress IFN production in herpesvirus infections,^62^ and anti-oxidants have been revealed to diminish viral activity.^63^^,^^64^ In our data, we observed the down-regulation of oxidative phosphorylation in bystander cells versus both infected and mock-control cells, particularly in Ciliated-3 cells (Figues 3B and 4A). This suggested that the down-regulation of these genes may occur as a result of IFN-stimulation, which is then reverted to original levels by viral factors. Down-regulation of oxidative phosphorylation may lead to the reduction in ATP in the host cell, required for viral processes in positive strand viruses,^65^^,^^66^ as well as a reduction of oxidative stress, thus providing an anti-viral state. IFNs have been shown to down-regulate mitochondrial genes in mouse NIH3T3 and Daudi lymphoblastic cells without viral infection,^67^ supporting this hypothesis. By using single-cell sequencing to separate infected and bystander cells, we have been able to identify that both up- and down-regulation are occurring as part of a fine balance in the control of key metabolic processes by host and viral factors.

We note that the main results of our study are based on the upper respiratory tract, namely, the nasal epithelia as it is the primary site of SARS-CoV-2 infection. However, SARS-CoV-2 has also been known to infect the lower respiratory tract, such as the lungs.^68^ The presence of ACE2 receptors required for host cell entry decreases from proximal to distal airways.^69^ In contrast to the upper airways, where ciliated cells are preferentially infected (as seen in our data – Figure 2E), the type II alveolar cells are most targeted by SARS-CoV-2 in the lower airways. As reviewed by Bridges et al.,^70^ *in vitro* studies involving human type II alveolar cells showed that there is a more rapid and robust immune response to SARS-CoV-2 than cells of the upper airway epithelia. Also, 3D organoids of the human airway epithelia have been utilized as models for SARS-CoV-2.^71^ Instead of a pseudo-stratified layer of cells like within ALI-cultures, these cells maintain the 3D structure that would be more akin to an actual *in vivo* airway epithelia. Interestingly, within 3D organoids from the lung, “immune-primed” subsets of ciliated and basal cells which exhibited higher expression of immune and inflammatory responses were observed.^23^ In contrast, our “immune-primed” subsets (i.e., +ISG^hi^) belonged to secretory and goblet cells (Figure 2A). Furthermore, nasal swabs from patients with COVID-19 showed an “IFN-γ-responsive” group of ciliated cells, which again contrasts with our ALI-HNEC data. Thus, these differences suggest that there are contrasting cellular effects of SARS-CoV-2 infection potentially driven by the methodology and/or location of the host cells along the respiratory tract.

Collectively, we highlighted the effectiveness of ALI-cultures as *in vitro* models for interrogating host-pathogen interactions and their usefulness for recapitulating SARS-CoV-2 infections.^17^^,^^29^^,^^72^ The heterogeneity of these cultures is vital for assessing cell-type-dependent responses to infection. Moreover, the coupling of scRNA-seq allowed the visualization of responses in different cell states (i.e., infected vs. bystander). These results suggested the importance of cellular metabolism during SARS-CoV-2 infections, and potentially the interplay between host responses (as shown in bystander cells) and viral hijacking of host processes (as shown in infected cells). This work may be extended via incorporating further age level categories with an increased number of donors, the addition of recent SARS-CoV-2 strains, and an extensive comparative study between various human airway epithelial ALI-culture-derived public datasets.

### Limitations of the study

Whilst we have utilized a pseudo-stratified airway model approach for our study which is superior compared with continuous clonal cell lines, we acknowledge that these results may not directly translate to *in vivo* situations. Particularly, the absence of immune cells in the model may distort the relevance of these results within this study. However, via this model, we were able to determine the epithelial immune responses to SARS-CoV-2 without confounding effects from immune-epithelial cell interactions. Additionally, we have also used a low MOI of <0.02, which may be more clinically relevant as low numbers of virions may initiate an *in vivo* infection. However, this means that the infection stage of each cell will be temporally asynchronized. The study is also limited to the small number of donors included per age group (three), limiting the robustness of the conclusions. As such, this study should be further extended with an increased number of donors. Despite this, the comparison between the strains is robust, as the background is tightly controlled using the same set of donors. In our results, one child donor (donor 6) showed cellular and infection differences compared with other donors. However, most cell-types and clustering aligned with other donors (as shown by the clustering analysis), and this effect has been minimized by applying filters such as the minimum number of donor replicates and minimum number of cells for pseudo-bulking. Furthermore, due to ambient RNA being able to be encapsulated into 10X droplets, there is a potential overestimation of true viral RNA load in each cell. We have applied a threshold of 10 UMI viral counts per cell to be deemed as infected to account for the potential contamination, according to the empirical threshold found by Ravindra et al.^17^ This work may be further improved by including more recent strains of interest, such as those within the Omicron lineage.

## STAR★Methods

### Key resources table

**Table:** 

REAGENT or RESOURCE	SOURCE	IDENTIFIER
**Bacterial and virus strains**		

SARS-CoV-2, VIC01/Ancestral	Laboratory of Damian Purcell	hCoV-19/Australia/VIC01/2020
SARS-CoV-2, VIC17991/Alpha	Laboratory of Damian Purcell	hCoV-19/Australia/VIC17991/2020

**Biological samples**		

Nasal turbinate brushings	Laboratory of Shafagh Waters, isolated from patient	N/A

**Critical commercial assays**		

Chromium Next GEM Single Cell 3’ Kit v3.1	10X Genomics	Cat# 10X-1000269
Dual Index kit TT Set A	10X Genomics	Cat# 10X-1000215
Library Construction Kit	10X Genomics	Cat# 10X-1000190

**Deposited data**		

Raw RNA-seq data	This paper	BioProject: PRJNA956316
10X processed data and genotyping analysis	This paper	GEO: GSE241292
Scripts/code used for analysis	This paper	https://github.com/cjy-23/ALI_scRNA_seq_SC2
Human genome GRCh38/hg38	10X Genomics	refdata-gex-GRCh38-2020-A
SARS-CoV-2 genome	Ensembl	ASM985889v3, INSDC Assembly GCA_009858895.3, Jan 2020

**Software and algorithms**		

PLINK v1.9	Purcell et al.^79^	RRID:SCR_001757; https://www.cog-genomics.org/plink/1.9/
Minimap2 v2.24	Li^80^	RRID:SCR_018550; https://github.com/lh3/minimap2
Samtools v1.16.1	Li et al.^81^	RRID:SCR_002105; https://www.htslib.org
bcftools v1.15.1	Danecek et al.^82^	RRID:SCR_005227; https://www.htslib.org
Nextclade v.2.14.0	Aksamentov et al.^83^	https://github.com/nextstrain/nextclade
Cellranger v6.1.1	10X Genomics	https://github.com/10XGenomics/cellranger
CrossMap v0.5.4	Zhao et al.^85^	RRID:SCR_001173; https://crossmap.sourceforge.net/
Demuxlet v11022021	Kang et al.^86^	https://github.com/statgen/demuxlet
Seurat v4.0.5	Hao and Hao et al.^33^	RRID:SCR_016341; https://satijalab.org/seurat/
scTransform v0.3.3	Hafemeister & Satija^88^	RRID:SCR_022146; https://github.com/satijalab/sctransform
dittoSeq v1.0.2	Bunis et al.^87^	https://github.com/dtm2451/dittoSeq
ShinyGO v0.76	Ge, Jung and Yao^89^	RRID:SCR_019213; http://bioinformatics.sdstate.edu/go76/
Speckle v0.99.7	Phipson et al.^91^	https://github.com/Oshlack/speckle
edgeR v3.30.3	Robinson, McCarthy and Smyth^93^	RRID:SCR_012802; https://bioconductor.org/packages/release/bioc/html/edgeR.html
limma v3.44.3	Ritchie et al.^94^	RRID:SCR_010943; https://bioconductor.org/packages/release/bioc/html/limma.html
Kraken2 v2.1.2	Wood, Lu & Langmead^96^	https://github.com/DerrickWood/kraken2
Monocle3 v1.3.4	Trapnell et al.^34^	https://cole-trapnell-lab.github.io/monocle3/
velocyto v0.17.17	La Manno et al.^97^	RRID:SCR_018167; http://velocyto.org/
loomR v0.2.1.9000	Laboratory of Rahul Satija	https://github.com/mojaveazure/loomR
scVelo v0.3.2	Bergen et al.^37^	RRID:SCR_018168; https://scvelo.readthedocs.io/en/stable/
Graphpad Prism v10.2.0	GraphPad Software	RRID:SCR_002798; https://www.graphpad.com/
multiGO	Laboratory of Lachlan Coin	https://coinlab.mdhs.unimelb.edu.au/multigo3/?dir=multiGO_sc/

### Resource availability

#### Lead contact

Further information and requests for resources and reagents should be directed to and will be fulfilled by the lead contact, Lachlan Coin (lachlan.coin@unimelb.edu.au).

#### Materials availability

This study did not generate new unique reagents.

#### Data and code availability

• Raw sequencing data have been deposited at Sequence Read Archive (SRA) and are publicly available as of the date of publication (BioProject: PRJNA956316). The DNA genotyping data reported in this study cannot be deposited in a public repository because of ethical restrictions. In addition, the processed 10X matrix information and the results of the genotyping data analysis have been uploaded to Gene Expression Omnibus (GEO) and are publicly available as of the date of publication (GEO: GSE241292). Accession numbers are also listed in the key resources table.
• All code has been deposited on Github and is publicly available as of the date of publication (https://github.com/cjy-23/ALI_scRNA_seq_SC2). The link is also listed in the key resources table.
• Any additional information required to reanalyze the data reported in this paper is available from the lead contact upon request.

### Experimental model and study participant details

#### Primary cultures of human origin

Human ethics permission was received from the Sydney Children’s Hospital Network Ethics Review Board (HREC/16/SCHN/120) and the Medicine and Dentistry Human Ethics Sub-Committee, University of Melbourne (HREC/2057111).^72^ Written consent was obtained from all participants (or participant’s guardian) prior to collection of biospecimens. All samples were de-identified before tissue processing. Culturing was carried out based on existing human nasal epithelial cell culture methods.^72^^,^^73^^,^^74^^,^^75^^,^^76^ Three healthy adult (PDI-5 (Male/32Y), PDI-1 (Female/32Y), and PDI-4 (Male/26Y)) and child (PDI-8 (Female/12Y), PDI-9 (Female/13Y), and PDI-10 (Male/14Y)) biobanked cells were utilized. Nasal turbinate brush samples were taken before the COVID-19 pandemic, ensuring no subject encountered SARS-CoV-2 exposure prior to *in vitro* infection. To initiate differentiation of ALI-cultures, cryo-preserved cells were thawed and seeded on to 6.5 mm Transwell inserts (Corning) pre-coated with PureCol-S collagen type I (Advanced BioMatrix). The cells were incubated at 37°C and 5% v/v CO_2_ until confluency in PneumaCult™-ExPlus media (STEMCELL Technologies) for 4-7 days before being switched to ALI-culture conditions by removing the apical media and feeding the basal side with PneumaCult™ ALI medium (STEMCELL Technologies). The cultures were incubated for 3-4 weeks to achieve mucociliary differentiation evidence by the presence of mucus and beating cilia. An analysis on the influence of sex on the results of our study was not performed due to the small sample size for each sex. However, any effects due to sex were accounted for in our models during DE analysis (see Quantification and statistical analysis).

### Method details

#### SARS-CoV-2 propagation and ALI-culture infections

Two strains of SARS-CoV-2 were utilized for this study – hCoV-19/Australia/VIC01/2020 (WT) and hCoV-19/Australia/VIC17991/2020 (Alpha). The propagation of the virus was carried out in Vero (African green monkey kidney epithelial – ATCC: CCL-81) cells cultured in MEM (MP Biomedicals), supplemented with 1 μg/mL TPCK-Trypsin (Trypsin-Worthington), penicillin (100 IU/mL), HEPES, Glutamax (Gibco), and streptomycin (100 IU/mL) under 37°C and 5% v/v CO_2_ incubation.^72^ Supernatant was harvested at 72 hpi, clarified via low-speed centrifugation before being filtered using a 0.45 μm syringe filter, aliquoted and stored at -80°C until use. Infectious titers were calculated by titration in Vero cells and the TCID_50_/mL was calculated using the Reed and Muench formula.^77^ All viral *in vitro* infections were performed in a BSCII in the BSL-3 laboratories located at the Peter Doherty Institute. Passages 4 and 3 of the WT and Alpha-variant stocks were used to infect the ALI-cultures, respectively.

The viral stock titers for the WT/VIC01 and Alpha/VIC17991 were 10^5.^^26^ TCID_50_/mL and 10^5^^.^^14^ TCID_50_/mL, respectively. 30 μL total inoculum was used in all cases, so the MOI is the maximum that could be achieved based on the virus stock with the lowest titer (Alpha/VIC17991). The equivalent amount of VIC01 was added. Overall, ALI-culture infections were carried out with an MOI of 0.014 in 30 μL of inoculum per insert (assuming ∼300,000 cell at the surface).^73^ After virus adsorption for 2 h at 37°C, the inoculum was washed off two times in total with PBS containing calcium and magnesium (PBS++), whereby this is the time at which 0 hpi is defined. At each timepoint (0, 24, 48, 72 hours post infection (hpi)), 200 μL of PBS++ was added to the apical surface and harvested after 10 min at 37°C before being stored at -80°C.

#### Viral stock sequencing

As titers and phenotypic presentation under microscopy may be influenced by adaptive mutations caused by passaging SARS-CoV-2 stocks in Vero-derivative cells (Vero E6),^78^ the viral stocks used for the infections were tested for mutations compared with the reference Global Initiative on Sharing All Influenza Data (GISAID) sequences. RNA was extracted using the QIAamp Viral RNA Mini Kit (Qiagen) using ∼0.5 mL of stock aliquot per column. A total of 3 x aliquots of VIC17991 and 2 x aliquots of VIC01 were extracted with 4 x 40 μL nuclease-free water in separate tubes without carrier RNA per column. After quantification with Qubit RNA High Sensitivity (HS) Assay Kit (Invitrogen), all the remaining RNA was pooled per strain. The pooled RNA was concentrated and cleaned using RNAClean XP beads (Beckman Coulter), with 2 x ethanol washes. The eluted RNA was diluted 1:10 and sequencing was performed using ONT Rapid Barcoding Kit 96 (SQK-RBK110.96) with the Midnight Primer Set (EXP-MRT001) supplied by ONT. Samples were sequenced on the ONT GridION with R9.4.1 flow cells with *MinKNOW* v23.07.12 for the GridION device and live-basecalling.

#### Immunofluorescence and confocal microscopy

Immunofluorescence and confocal microscopy imaging was performed as previously described.^72^ In brief, the cells were washed three times with PBS++ at the time of harvest (72 hpi). Mock-control cells were harvested at 7 days post-infection (dpi). Cells were then fixed immediately with 4% paraformaldehyde (#15710, Electron Microscopy Sciences, USA) for half an hour at room temperature. The fixative was removed and replaced with 100 mM glycine in PBS++ for 10 minutes to neutralize the remaining fixative. Cells were permeabilized with 0.5% Triton-X in PBS++ for half an hour on ice, before being washed 3 times with PBS++ at room temperature. At this stage, the membranes were carefully excised from the Transwell inserts, cut into half, one for test antibodies and the other for control antibodies, and blocked for 90 minutes at room temperature in immunofluorescence (IF) buffer (PBS++ with 0.1% bovine serum albumin, 0.2% Triton, 0.05% Tween 20) supplemented with 10% goat serum. After this, the block buffer was replaced by block buffer containing the primary antibodies, anti- acetylated α-tubulin (Sigma-Aldrich #T7451, diluted at 1:250) and anti-SARS Nucleocapsid Protein (Novus Biologicals #NB100-56683, diluted at 1:200). After incubation for 48 hours at 4^o^C, the primary antibodies were washed off with IF buffer 3 times; then, fluorophore conjugated secondary antibodies, goat-anti-mouse Alexa Fluor 488 (Invitrogen #A11001) and goat-anti-rabbit Alexa Fluor 647 (Invitrogen #21244) and Hoechst, were added and incubated for 3 hours at room temperature in the dark. Secondary antibodies were then washed off 5 times with IF buffer. The membranes were incubated with DAPI for half an hour, washed once with PBS++ and transferred to slides where they were mounted in FluoroSave Reagent (#345789 EMD Millipore). The confocal microscopy imaging was acquired on the Zeiss LSM 780 system. The acquired images were processed using *ImageJ* software.

#### 10X Genomics single-cell preparation

The ALI-cultured HNECs were prepared for the 10X Chromium step according to the Single Cell Protocols Cell Preparation Guide General Sample Preparation RevC (10X Genomics). Briefly, cells were dissociated using trypsin and filtered through a 40 μm strainer and pipette-mixed to ensure a single-cell suspension. The cells were washed with PBS with 0.04% BSA. Once cells were counted, they were harvested for mock-infected control, 48 hpi (WT infection) and 72 hpi (WT and Alpha variant infection) conditions for each donor (per age-group) and used as input for the 10X Chromium preparation. The Chromium Single Cell 3ʹ Reagent Kit v3.1 (10X Genomics) was utilized in conjunction with Dual Index kit TT Set A barcodes (10X Genomics) for multiplexing.

#### Illumina RNA-sequencing

Each Illumina library was quantified with Qubit 4.0 Fluorometer via the Qubit 1X dsDNA HS Assay Kit (Invitrogen), and the fragment sizes were tested with Tapestation 4200 (Agilent Technologies) using the High Sensitivity D5000 ScreenTape (Agilent Technologies). All libraries were pooled according to respective molarities. The pooled libraries were split equally and sequenced on three NovaSeq S4 2x150bp lanes using the NovaSeq kit v1.5 (Illumina) with 0.5% of PhiX via the NovaSeq 6000 Sequencing System. The cycling parameters were as follows: Read 1 – 150 bp, Index 1- 10 bp, Index 2 – 10 bp, Read 2 – 150 bp. The sequencing was carried out by Ramaciotti Centre for Genomics at the University of New South Wales (UNSW). A total of ∼7.95 billion reads were acquired.

#### Genotyping

Genotyping for each of the donors was required to accurately demultiplex the mixed population of cells used as inputs into the 10X Chromium preparation. DNA extraction for genotyping was carried out with the DNeasy Blood and Tissue Kit (Qiagen) according to the manufacturer’s guidelines (Purification of Total DNA from Animal Blood or Cells (Spin-Column Protocol)) with minor modifications. Briefly, ALI-culture membranes were excised from the inserts and placed into tubes containing PBS and proteinase K. Once Buffer AL was added, the sample was briefly vortexed and incubated at 56°C for 10 minutes with a Thermomixer C (Eppendorf) at 1000 rpm. 100% ethanol was added to the reaction and tubes briefly vortexed. The reaction was loaded on to a spin-column and all subsequent spins were carried out at 12,000 rpm except for step 6 of the protocol, where after adding AW2 buffer, the columns were spun at 14,000 rpm. 200 μL of buffer AE was used to elute the DNA and passed through the column in total of three times to concentrate the sample. Quality control of DNA was carried out using Qubit 4.0 Fluorometer via the Qubit 1X dsDNA HS Assay Kit, BioAnalyzer 2100 using the High Sensitivity DNA Assay and NanoDrop 2100 Spectrophotometer (ThermoFisher Scientific). Genotyping was carried out using DNA derived from each individual donor using the Infinium Global Screening Array (GSA) v2.0 BeadChip (Illumina) and performed by Macrogen (Korea). The reference annotation used was GRCh37.

### Quantification and statistical analysis

#### Viral titer analysis

Statistical analyses and graphing of TCID_50_ results from apical washes at 0, 24, 48, 72 hpi comparing adults and children with WT and Alpha infections were carried out using *GraphPad Prism* v10.2.0. Data are represented as mean log virus titer ± SD, n=3, where each n represents a donor within an age-group (adult/child), with each n averaged from 1-3 technical replicates. Statistical testing was carried out with two-tailed paired T-test for WT vs Alpha analyses within each age-group and Welch’s T-test for adult vs child analyses (p = ∗ ≤ 0.05, ∗∗ ≤ 0.01). Only significant values are notated (p ≤ 0.05). The statistical results can be found in Figure 1A.

#### Genotyping analysis

*PLINK* v1.9^79^ was utilized to convert the output of the genotyping data (from section Genotyping) to VCF files. Firstly, the sex of the samples was checked, and this information was incorporated into the data. Heterozygous haploid hardcalls and all female chrY calls were erased from the data. The resulting file was converted to VCF file with ‘–recode’. Variants with one or more multi-character allele codes and single-character allele codes outside of {‘A’, ‘C’, ‘G’, ‘T’, ‘a’, ‘c’, ‘g’, ‘t’, <missing code>} were removed from the data. To match chromosome names with downstream processes, ‘chr’ was added to the chromosome names, all rows with ‘chr0’ was removed, then chromosomes were arranged lexicographically. The final file was gzipped by *bgzip* and indexed via *tabix*.

#### Viral stock variant analysis

The passed and trimmed reads from ONT sequencing were mapped to corresponding references using *Minimap2* v2.24^80^ and *Samtools* v1.16.1^81^ with the long sequence option. The output BAM files and the references were input to create consensus genomes with *bcftools* v1.15.1.^82^ Then, the consensus genomes were analysed using *Nextclade* v2.14.0^83^ with SARS-CoV-2 database (reference number MN908947) to call mutations.

The WT viral stock used for the infections contained an additional S:H655Y amino acid substitution compared with the original VIC01 strain GISAID sequence, which has shown to cause increase in infectivity.^84^ Furthermore, the Alpha viral stock contained a longer genome (29,901 nt) sequence than the reference VIC17991 strain GISAID sequence (29,767 nt), which subsequently led to an additional deletion of G113 in ORF1a and a reduction of deletions annotated with the VIC17991 GISAID sequence (Table S4).

#### Illumina data processing

BCL files from Illumina sequencing were converted to FASTQ files using *Cellranger* v6.1.1 (10X Genomics) with the ‘mkfastq’ function. Count files were produced with *Cellranger* ‘count’ using the reference package ‘refdata-gex-GRCh38-2020-A’. BAM files generated from GRCh38/hg38 were lifted with liftover tool *CrossMap* v0.5.4^85^ to GRCh37/hg19 to enable incorporation of the donor genotype information which was analyzed using GRCh37. Similarly, a *Cellranger* reference was created for SARS-CoV-2 reference genome using *Cellranger* ‘mkref’ using the Ensembl reference ASM985889v3, INSDC Assembly GCA_009858895.3, Jan 2020 and a custom GTF file by setting the whole genome as an exon. Viral counts were determined separately but similarly to host counts using *Cellranger* ‘count’. For the viral counts, raw matrices instead of filtered matrices were utilized for downstream analysis as the effect of filtered matrices (i.e. filtering of artifactual cells) was not applicable to the viral counts. The VCF files and the sorted GRCh37 BAM files were used as inputs to *Demuxlet* v11022021.^86^ This enabled the donor assignment to cell barcodes and estimation of the number of doublets in the data.

#### Data filtering and unsupervised clustering

For downstream analysis of Illumina datasets, *Seurat* v4.0.5 was implemented.^33^ *Seurat* objects were created separately for both viral and host counts data. The demultiplexing information from *Demuxlet* was incorporated into the *Seurat* object using *importDemux* from *dittoSeq* v1.0.2.^87^ Firstly, the viral data was separated based on infection tier as follows; uninfected <10, low =10-99, medium =100-999, high =1,000-9,999, very high ≥10,000 UMI counts. This information was incorporated into host meta data per cell-barcode. Uninfected cells (<10 UMI counts) with exposure to virus were assigned as ‘bystander’ cells. Then, the data was filtered for singlets, according to the *Demuxlet* results. Cells which had <20% mitochondrial RNA, >5% ribosomal RNA were kept for analysis. Also, cells with greater than 200 and less than 9000 detected genes were kept, and genes expressed in at least 3 cells were kept for analysis. Each of the 16 libraries (i.e. 8 x main and 8 subsample) were handled separately. Data was normalized and scaled using *scTransform* v0.3.3^88^ (using the original method) and differences in cell cycle were regressed out by the alternate workflow (regressing out the G2M – S phase scores). Then, these datasets were merged using *Seurat* ‘merge’. Utilizing cell markers from scRNA-seq datasets and within the literature,^17^^,^^31^^,^^32^ unsupervised clustering was performed. ‘FindAllMarkers’ was used to detect cell markers, with default parameters (i.e. min.pct=0.1, logfc.threshold=0.25, Wilcoxon’s test of ranks). Final parameters used were dims=1:20 for ‘RunUMAP’ and ‘FindNeighbors’ and resolution=0.3 for ‘FindClusters’ functions. Characteristics of sub-cell-types (e.g. Ciliated 1-3 among ciliated cells) have been analyzed by running the ‘FindMarkers’ function via *Seurat,* which uses the Wilcoxon’s test of ranks with Bonferroni *p*-value adjustment. Parameters of min.pct=0.25 and min.diff.pct=0.25 were used. Subsequently, using *ShinyGO* v0.76,^89^ we assessed the enriched gene ontology (GO) biological pathways of significantly DE genes (p_adj_ < 0.05), via the option “Select by FDR, sort by Fold Enrichment” with the default background list.

In our results, one child donor (donor 6) showed notable differences to other donors. Although largely similar to the other donors, in uninfected cultures, the clustering data showed the expansion of goblet cells, and a separated group of basal (Basal-2), secretory (Secretory-2) and ciliated (Ciliated-2) cells. This was also observed in the other treatment datasets (WT 48, WT 72 and Alpha 72 hpi) (Data S2). Physically, the cells were larger and more oval in shape compared with the other two child donors with atypical morphology (Figure S2A). To understand the reasoning behind the differences observed in this donor at a deeper level, we tested for 1) epithelial-mesenchymal transition (EMT) using the marker Vimentin (*VIM*) (Figures S10A and S10B), 2) metagenomics using *Kraken2Uniq*^90^ to rule out any asymptomatic co-infections within this donor (Data S5, see Immune profiles & test for EMT), and 3) immune-state differences between mock-control cells (Figure S10C, see Immune profiles & test for EMT). However, overall, the results did not show robust evidence of these aforementioned effects contributing to the lack of infection or separation of cell clusters in this donor in comparison to other donors. As the clustering showed substantial overlap with the other donors, we proceeded with the analysis with the inclusion of this donor.

#### Cell proportion change testing

The changes cell-type proportions for each treatment condition (Alpha 72, WT 72 and WT 48 hpi) were tested using *Propeller* via *Speckle* v0.99.7^91^ via the ‘propeller.ttest’ function in *R* v4.2.0. Each donor was used as replicates, and the tests were carried out within the same age-group (adult/child) between the mock-infected control datasets and each infected dataset (Alpha 72, WT 72 and WT 48 hpi). The data are represented as mean with n=3, where each n is each donor within each age-group (adult/child, p = ∗ ≤ 0.05, ∗∗ ≤ 0.01, ∗∗∗ ≤ 0.001, ∗∗∗∗ ≤ 0.0001). The percentages of each cell-type in mock-control cells vs infected datasets were plotted using *ggplot2* v3.4.0. The statistical results are found in Figure S3 & Data S4. The percentages of secretory-ciliated cells within each age-group between conditions were carried out using the two-tailed paired T-test with n=3 ± SD, where each n is each donor within each age-group (adult/child, p = ∗ ≤ 0.05, ∗∗ ≤ 0.01). The statistical results are found in Figure 2F.

#### Differential gene expression (DGE) analysis

After identifying the different cell-types, DGE analysis was performed on the host via a pseudo-bulking method. Briefly, the data was separated, and the counts were aggregated by a unique combination of cell-type, treatment, age-group, infection status and donor information. All samples were filtered by a minimum of 15 cells and genes which had counts in less than 10 cells were removed from the analysis. For DGE analysis, each comparison was carried out by including samples which had at least two donor replicates on each side of the comparison. Following the results from Squair et al.^92^ the *edgeR-*likelihood ratio test (edger-LRT) method was carried out on the aggregated counts via *edgeR* v3.30.3.^93^ The effect of sex was added into the linear model to account for sex-effects. The *p*-values were adjusted via the Benjamini-Hochberg method. We note that any comparisons between Ciliated+SARS^hi^, Secretory-Ciliated+SARS^hi^, Secretory+ISG^hi^, Goblet+ISG^hi^ and mock-control dataset was compared with controls from Ciliated-1, Secretory-Ciliated, Secretory-1&2 and Goblet clusters, respectively, due to lack of control cells in the clusters. The populations of matching control cells were determined by closest cell states. GO biological and reactome pathways were visualized using an in-house visualization tool – *multiGO* (Table S3). The parameters used were pv_thresh=0.05, enrichment pv_thresh=0.005 and logFC_thresh=1. Background lists of genes were curated from all genes tested for DGE in all groups which were displayed in each *multiGO* analysis via setting the ‘Background set for DE’ parameter as ‘gene_list’ (Table S3).

#### Immune profiles & test for EMT

As donor 6 showed lower viral load (Figures S1B and S1D) and showed some differences in cellular composition to other donors (Figures S2A and S2E–S2G), we investigated the differences in immune profiles and the expression of EMT marker *VIM* for donor 6 without infection. DE was performed using a similar pseudo-bulking approach as the main DE analysis. Firstly, all mock-control cells from donor 6 were compared against the other two child donors, and then between all other donors as a bulk analysis. This was carried out via the limma-voom method through *limma* v3.44.3^94^ and *edgeR* v3.30.3.^93^ The differences in genetic variability between donors were regressed out blocking the ‘batch’ (i.e. each donor) factor variable as a random effect. The effect of sex was added as a fixed effect in the design matrix. GO biological and reactome pathways were visualized using an in-house visualization tool – *multiGO*. The parameters used were pv_thresh=0.05, enrichment pv_thresh=0.005 and logFC_thresh=1. Background lists of genes were curated from all genes tested for DE in all groups which were displayed in each *multiGO* analysis via setting the ‘Background set for DE’ parameter as ‘gene_list’ (Table S3).

#### Asymptomatic co-infection testing

Following the reasons described in the previous section (Immune profiles & test for EMT), we next wondered whether this difference observed in donor 6 was due to an asymptomatic co-infection. The analysis was carried out using a metagenomic testing approach. The output BAM files from mock-infected child donor datasets from the larger group (85% cells, ‘Short_read_uninfected_child’) which were mapped to the human genome from *Cellranger* were demultiplexed with data from *Demuxlet*. This was carried out via isolating singlet cell barcodes matching to each of the child donors and extracting the data using *Samtools* v1.9. The demultiplexed files were then filtered for unmapped reads using *Samtools* ‘view -b -f 4’ to deplete already human-mapped reads. The unmapped reads were converted back into paired-end FASTQ format using *Cellranger*’s ‘bamtofastq’ function. Reads [from the depletion step] were classified using *Kraken2Uniq* protocol for pathogen detection with a minimum hit groups setting of 3^90^^,^^95^ and the PlusPF database based on RefSeq (2022-09-08, https://benlangmead.github.io/aws-indexes/k2). Taxonomic classification reports were inspected manually for the presence of viral, bacterial and eukaryotic taxa that may cause co-infections, evaluating abundance, number of reads classified and the number of distinct minimizers in relation to the number of reads.^90^ *Kraken2* v2.1.2^96^ was utilized for this analysis with the parameters ‘--db angmead_pluspf_64GB/ --minimum-hit-groups 3 --report-minimizer-data --threads 32--output ${name}.kraken2uniq --report ${name}.kraken2uniq.report’.

#### Trajectory analysis

To investigate putative developmental trajectories in the data, we used the *Monocle3* package v1.3.4.^34^ First, we down-sampled the data to make it more tractable for the trajectory algorithms, as well as more in line with any potential assumptions stemming from the typical size of a single-cell data set: Using the sample function in *R*, we randomly selected 10,000 cells as a subset from the original data. Following the *Monocle3* pipeline, we clustered the cells (‘cluster_cells’), learned the trajectory graph (‘learn_graph’), and ordered the cells (selecting the basal populations as starting point – ‘order_cells’). We then plotted the trajectories and pseudo-time on the UMAP reduction.

#### RNA velocity analysis

In order to better understand the trajectory between ciliated, secretory and secretory-ciliated cells, the *Seurat* object was subset to contain all relevant cell-types (i.e. Secretory-1, Secretory-2, Secretory+ISG^hi^, Ciliated-1, Ciliated-2, Ciliated-3, Ciliated+SARS^hi^, Secretory-Ciliated+SARS^hi^, Goblet+ISG^hi^, Goblet, Secretory-Ciliated). This object was then further split into a combination of ‘type’ (e.g. Long_read_UK_72hpi) and donor (e.g. Adult 1). ‘NormalizeData’, ‘FindVariableFeatures’, ‘RunPCA’ and ‘CellCycleScoring’ with alternate workflow to calculate the difference between S – G2M phases (CC.Difference) were applied to each individual object. Subsequently, *scTransform* was applied on each individual object (using the original method), with CC.Difference being regressed out during this process. The individual objects were merged, and clustering was carried out with dims=1:10 in ‘RunUMAP’ and ‘FindNeighbors’ and with resolution=0.3 in ‘FindClusters’ functions. Then, subclustering was carried out using the ‘FindSubCluster’ function. Using the ‘RNA’ assay, the data was renormalized using ‘NormalizeData’, ‘FindVariableFeatures’ and ‘ScaleData’. The cell-types were assigned using the same method as above in section ‘Data filtering and unsupervised clustering’.

Barcodes from subset *Seurat* objects were isolated and used to run *velocyto* v0.17.17^97^ along with the BAM files from the *Cellranger* output with the ‘genes.gtf’ file from the *Cellranger* reference package as inputs. *Samtools* v1.16.1 was also utilized in this process. The resulting LOOM files were converted to *Seurat* objects using *loomR* v0.2.1.9000 and the UMAP and PCA embeddings from the *Seurat* object in the aforementioned step were merged into the *Seurat* object converted from LOOM files. The data was downsampled to 10,000 cells and *scVelo* v0.3.2^37^ was used with the dynamical model to perform the RNA velocity analysis. As a part of the *scVelo* analysis, *PAGA*^98^ analysis was carried out to examine the directionality of trajectory with RNA velocity taken into account. The filtering and normalization was carried out with the ‘scv.pp.filter_and_normalize’ function with parameters min_shared_counts=20, n_top_genes=2000, and the ‘scv.pp.moments’ function with parameters n_pcs=30, n_neighbors=30.

#### Cytokine-related gene expression

To investigate the differences in the expression of cytokine-related genes in different infection levels per viral load, genes from the GO biological term (*cytokine production involved in immune response*) were isolated. The Alpha 72 hpi and WT 72 hpi data involving adults were subsetted using *Seurat’*s ‘subset’ function and further subsetted into bystander, low and high/very high infection levels. Using *Seurat*’s built-in ‘DotPlot’ visualization function, the average expression of cytokine-related genes were plotted.

## Supplemental information

Document S1. Figures S1–S10 and Tables S1–S4

Data S1. Output from *Seurat* FindAllmarkers to determine cell clusters, related to Figure 2A Default parameters were used.

Data S2. Percentage of each cell-type per donor in each condition (control, WT 48 hpi, WT 72 hpi, Alpha 72 hpi), related to Figure 2A

Data S3. Number and percentages of uninfected and infected cells in each condition (control, WT 48 hpi, WT 72 hpi, Alpha 72 hpi), related to Figure 2C

Data S4. Outputs from *Propeller* analysis of changes in cell-type distributions, related to Figure 2F

Data S5. Outputs from *Kraken2Uniq* analysis of uninfected donor 6 Illumina datasets, related to STAR Methods
