# Supplementary material for: Uncovering strain- and age-dependent innate immune responses to SARS-CoV-2 infection in air-liquid-interface cultured nasal epithelia
Source: iScience. 2024 May 17;27(6):110009. doi: 10.1016/j.isci.2024.110009 (PMC11166695; doi:10.1016/j.isci.2024.110009)
Supplement: Document S1. Figures S1–S10 and Tables S1–S4 [file mmc1.pdf]

iScience, Volume *27*

Supplemental information

Uncovering strain- and age-dependent innate immune responses to SARS-CoV-2 infection in air-liquid-interface cultured nasal epithelia

Jessie J.-Y. Chang, Samantha L. Grimley, Bang M. Tran, Georgia Deliyannis, Carolin Tumpach, An N.T. Nguyen, Eike Steinig, JianShu Zhang, Jan Schröder, Leon Caly, Julie McAuley, Sharon L. Wong, Shafagh A. Waters, Timothy P. Stinear, Miranda E. Pitt, Damian Purcell, Elizabeth Vincan, and Lachlan J.M. Coin

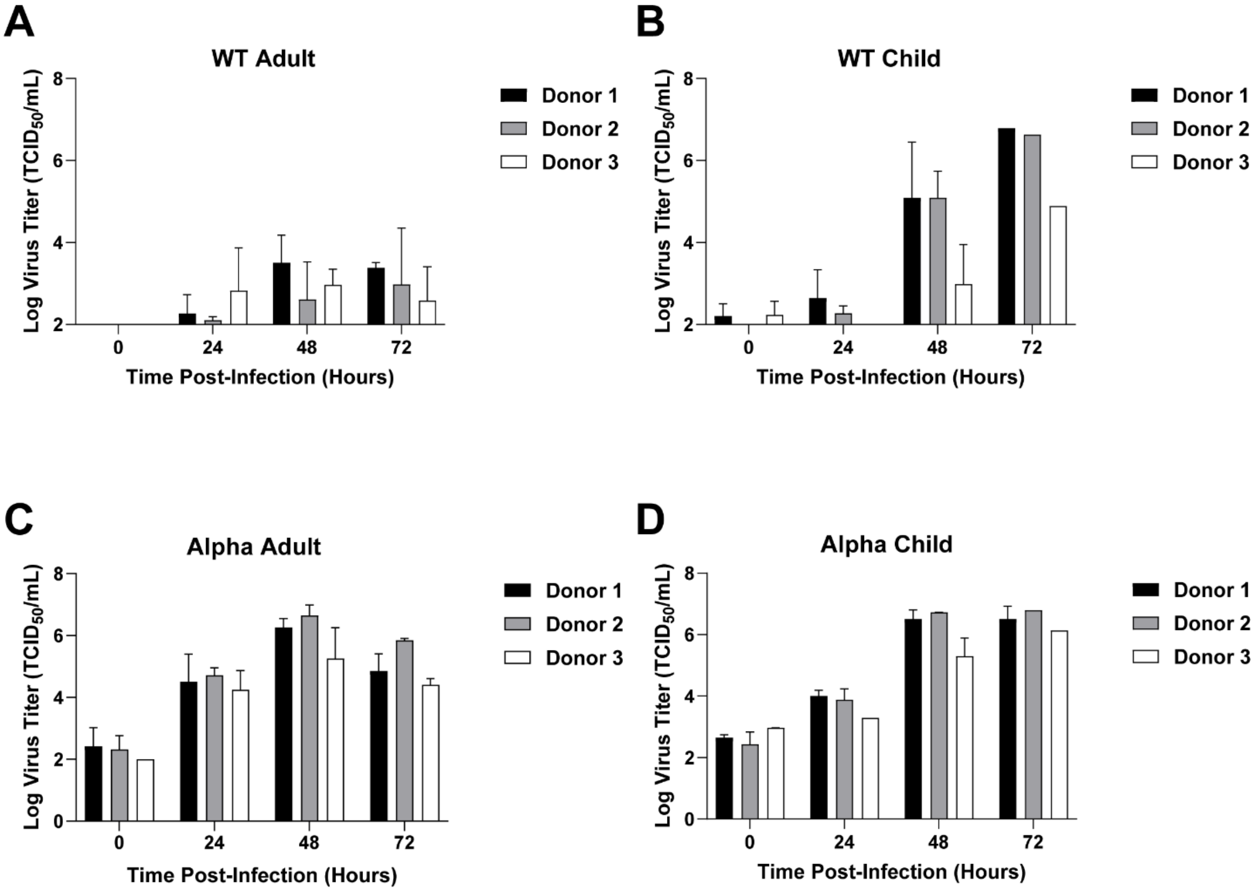

Figure S1. Viral titers of SARS-CoV-2 infected ALI-HNECs per donor show age-dependency, related to Figure 1a. (A-D) $TCID_{50}$ results from apical washes at 0, 24, 48, 72 hpi comparing **(A-B)** WT-infections in **A)** adults and **B)** children, and **(C-D)** Alpha-infections in **C)** adults and **D)** children. Data are represented as mean show log virus titer + SD, each bar averaged from 1-3 technical replicates.

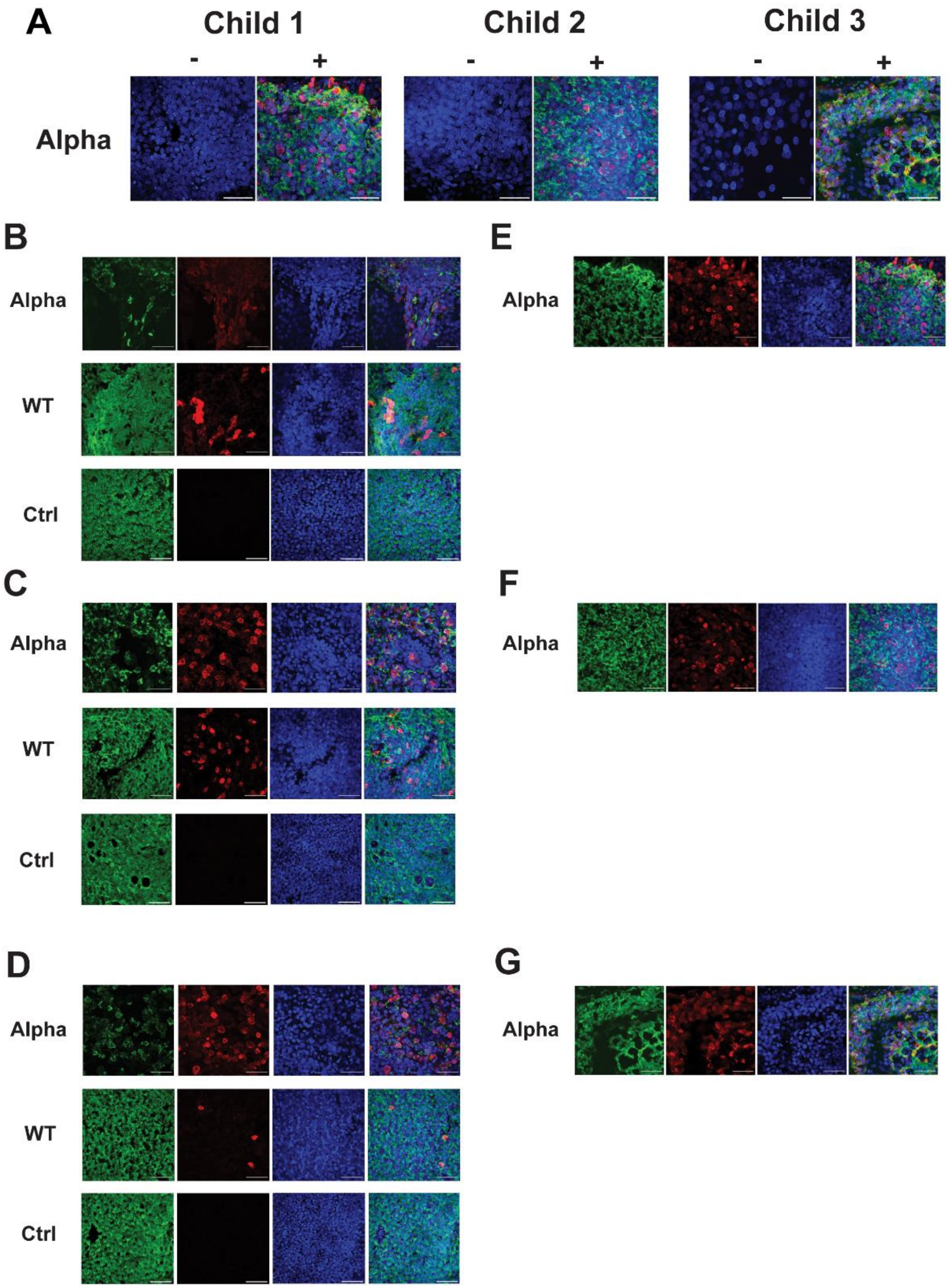

Figure S2. Immunofluorescent confocal microscopy staining at 40X magnification of ALI-HNECs reveals strain- and age-dependent loss of cilia, related to Figure 1b. A) Child ALI-cultures infected with Alpha-variant harvested and imaged at 72 hpi. **(B-G)** Individual channels shown with all donors with final combined channels. **(B-D)** Stains in adult donor cells infected with WT and Alpha SARS-CoV-2 in **B)** adult 1, **C)** adult 2, **D)** adult 3. **(E-G)** Stains in child donors infected with Alpha SARS-CoV-2 in **E)** child 1, **F)** child 2 and **G)** child 3. Stained for α-tubulin (AcTub, green), nucleoprotein (NP, red) and nuclei (DAPI, blue). WT-infected, Alpha-infected, and mock-control cells are shown for adults and only Alpha-infected cells are shown with children due to lack of spare ALIs available for children. Mock-control cells were harvested at 7 days post-infection (dpi). Scale bar: 50 µm.

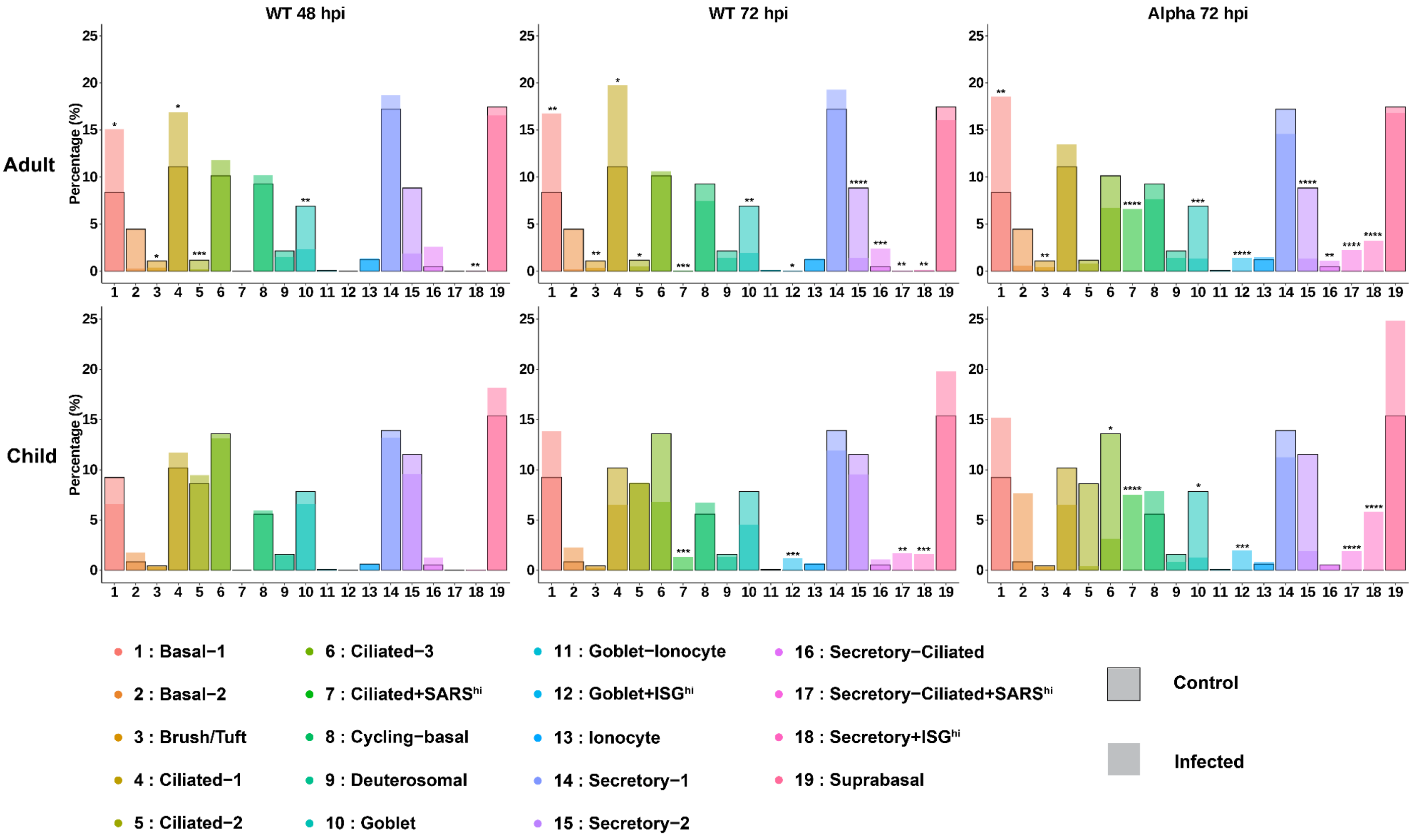

Figure S3. Percentages of each cluster in infected compared with mock-control datasets in adult and child ALI-cultures, related to Figures 2f-g. X-axis shows the number representing the various cell-type clusters in the data and the Y-axis shows the percentage contribution of these cell-types. Bars with black outlines indicate the percentage of cluster in the mock-control dataset, and the bars without an outline indicate the percentage of cell-type cluster in the infected dataset (i.e. WT 48 hpi, WT 72 hpi, Alpha 72 hpi). Data are represented as mean, n=3, where n is each donor within an age-group (adult/child, p = * ≤ 0.05, ** ≤ 0.01, *** ≤ 0.001, **** ≤ 0.0001, moderated T-test).

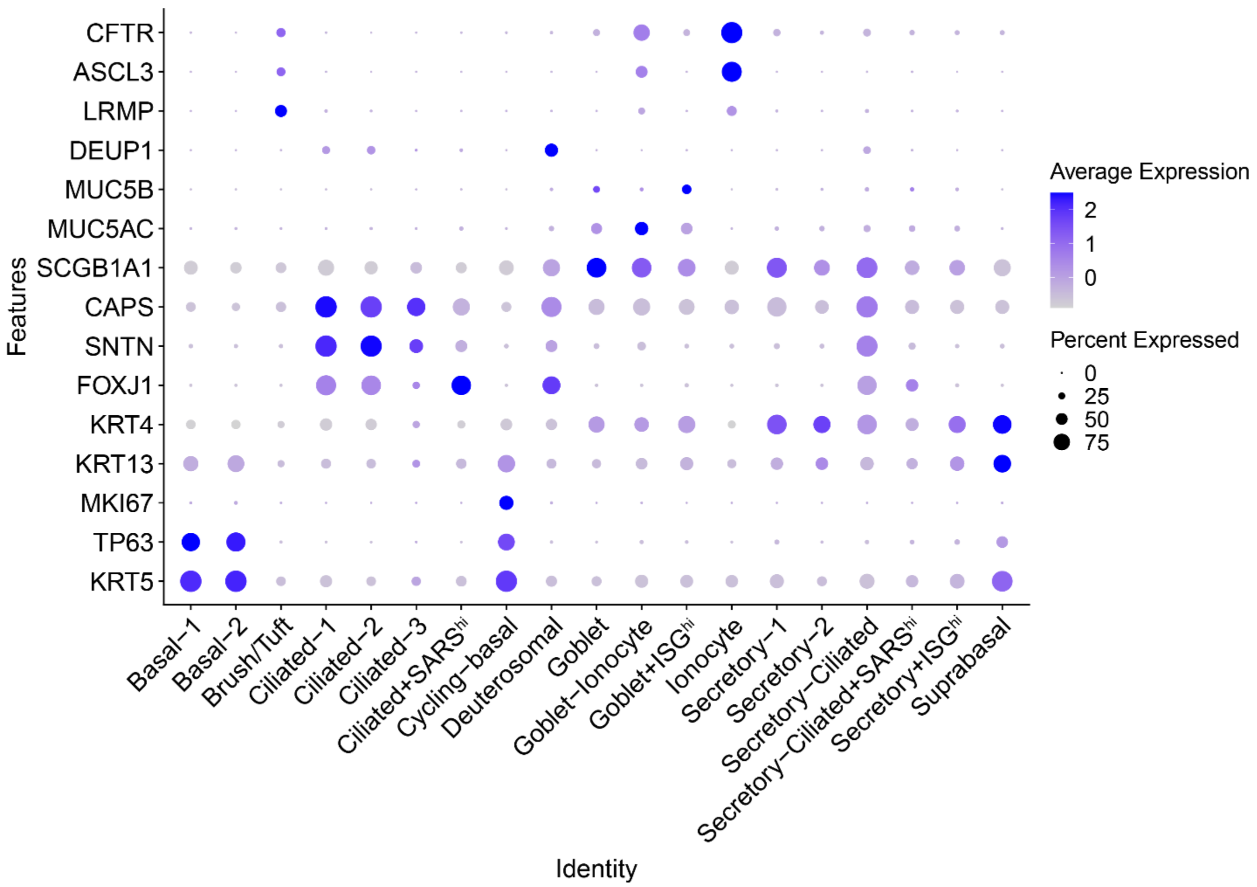

Figure S4. Dot plot showing the expression of major cell-type-specific markers in each cell-type, related to Figures 2a & f. X-axis shows the individual cell-types within the data, and the Y-axis presents the cell-type marker genes. Bubble size indicates proportion of cells in cell cluster expressing the gene, and color indicates the average expression.

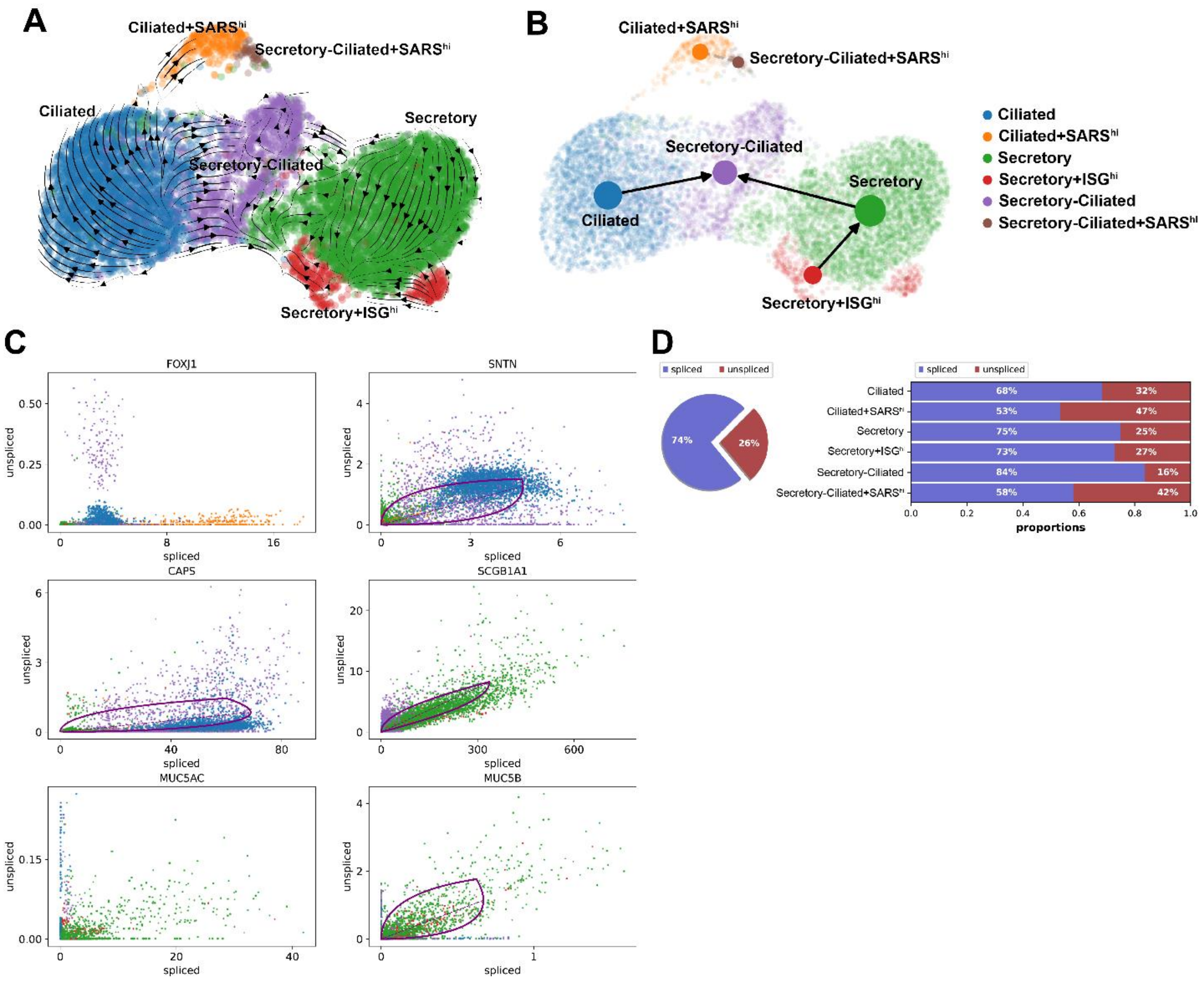

Figure S5. RNA velocity directionality reveals transcriptional dynamics in cells with secretory and/or ciliated properties, related to Figure 2b. A) RNA velocities (dynamical model) projected onto pre-computed UMAP embeddings. **B)** Partition-based graph abstraction (PAGA) graph shows directionality from Secretory/Ciliated → Secretory-Ciliated cells **C)** Phase portraits of marker genes for ciliated (*FOXJ1, SNTN, CAPS*) and secretory (*SCGB1A1, MUC5AC, MUC5B*) cells. X-axis represents the expression of spliced mRNA and Y-axis represents the expression of unspliced mRNA. **D)** Proportion of spliced (blue) and unspliced (red) transcripts in each cluster.

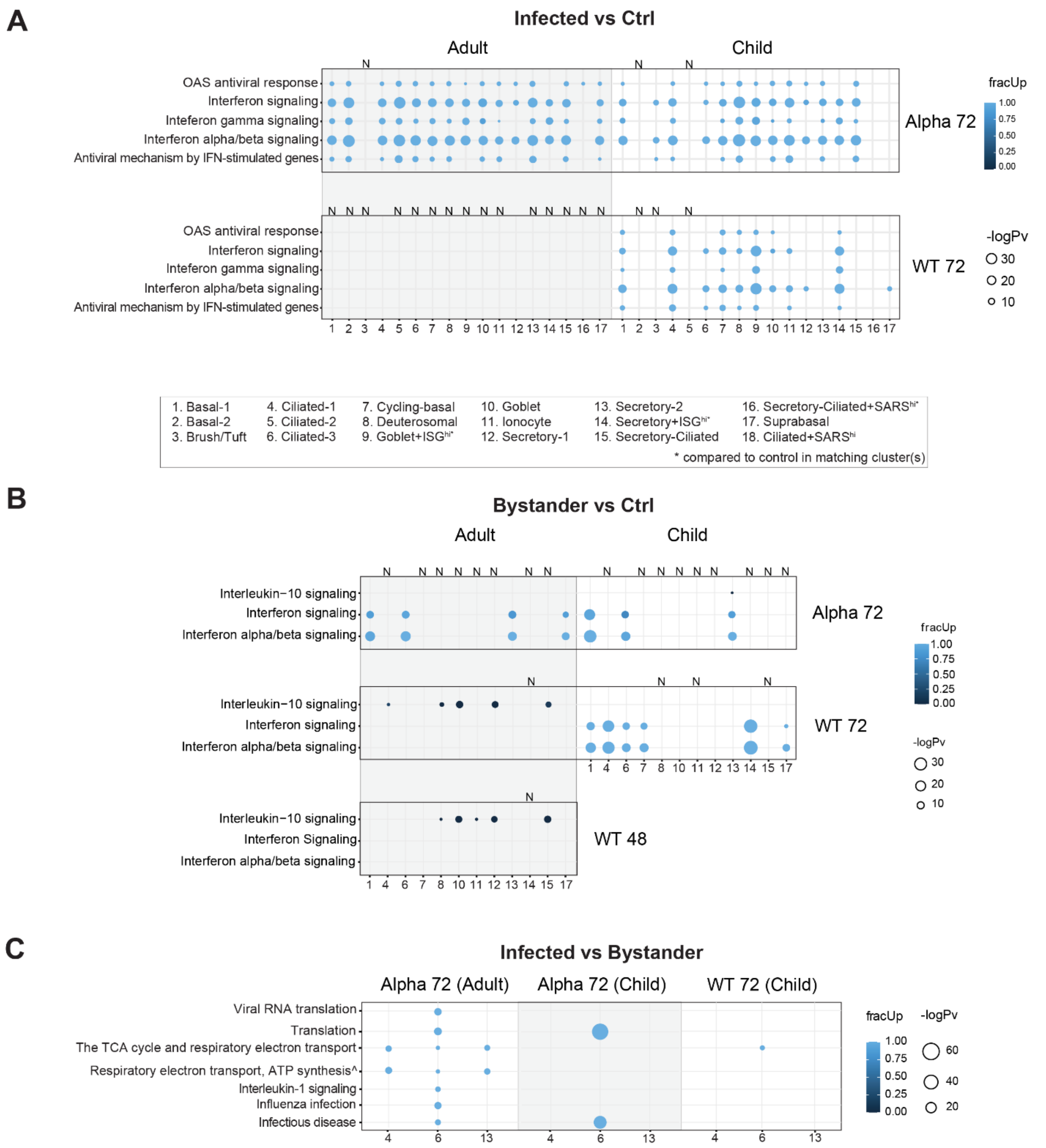

Figure S6. Significantly enriched reactome pathways analyzed using *multiGO* using significant DE results, related to Figures 3a-b & 4a. **A)** Infected vs mock-control cells, **B)** bystander vs mock-control cells and **C)** infected vs bystander cells. Columns with no matching DE data available are denoted with 'N'. Bubble size indicates -$\log_{10}$ enrichment p-values, and the color of the bubble indicates the proportion of upregulated genes involved in the pathway (i.e. fracUp). A subset of the results from top 35 terms are shown except for **Figure S6c** which shows the subset of results from top 100 terms. ^ abbreviation of *Respiratory electron transport, ATP synthesis by chemiosmotic coupling, and heat production by uncoupling proteins*. The same numbering of cell clusters shown in the legend are used across the panels. Thresholds of pv_thresh=0.05, enrichment pv_thresh=0.005 and logFC_thresh=1 were used. A full list of enriched reactome pathways is available via the links in **Table S3**.

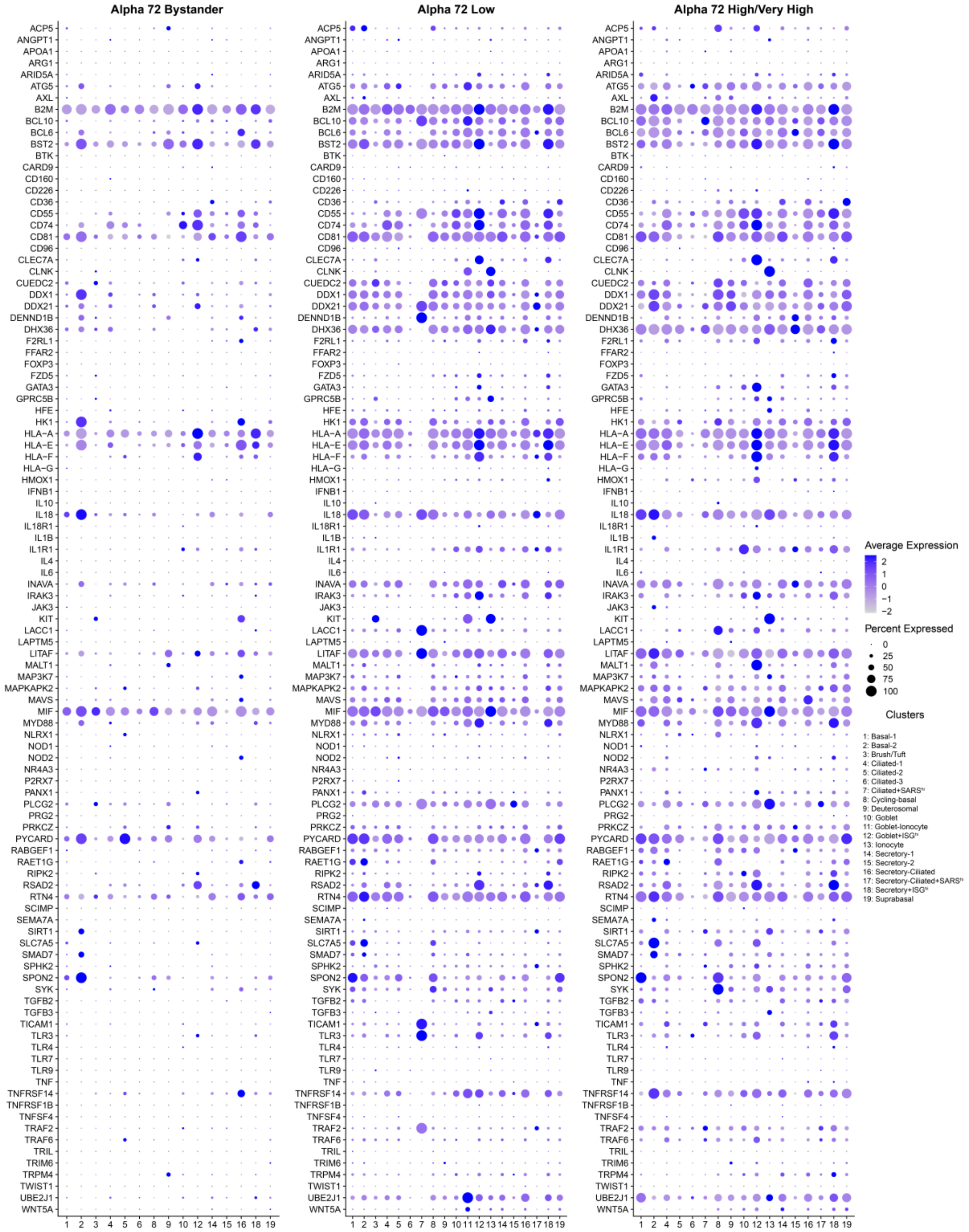

Figure S7. Dot plot showing average expression of cytokine-related genes involved in GO biological term (*cytokine production involved in immune response*) separated into bystander, low and high/very high infection categories in Alpha 72 hpi adult datasets, related to Figures 3a-b. X-axis shows the number representative of each cell-type cluster (shown in legend) and Y-axis shows the genes involved in the GO biological term (*cytokine production involved in immune response*). The color of the bubble represents the average expression level, and the size of the bubble represents the percentage of cells in each cluster expressing the gene.

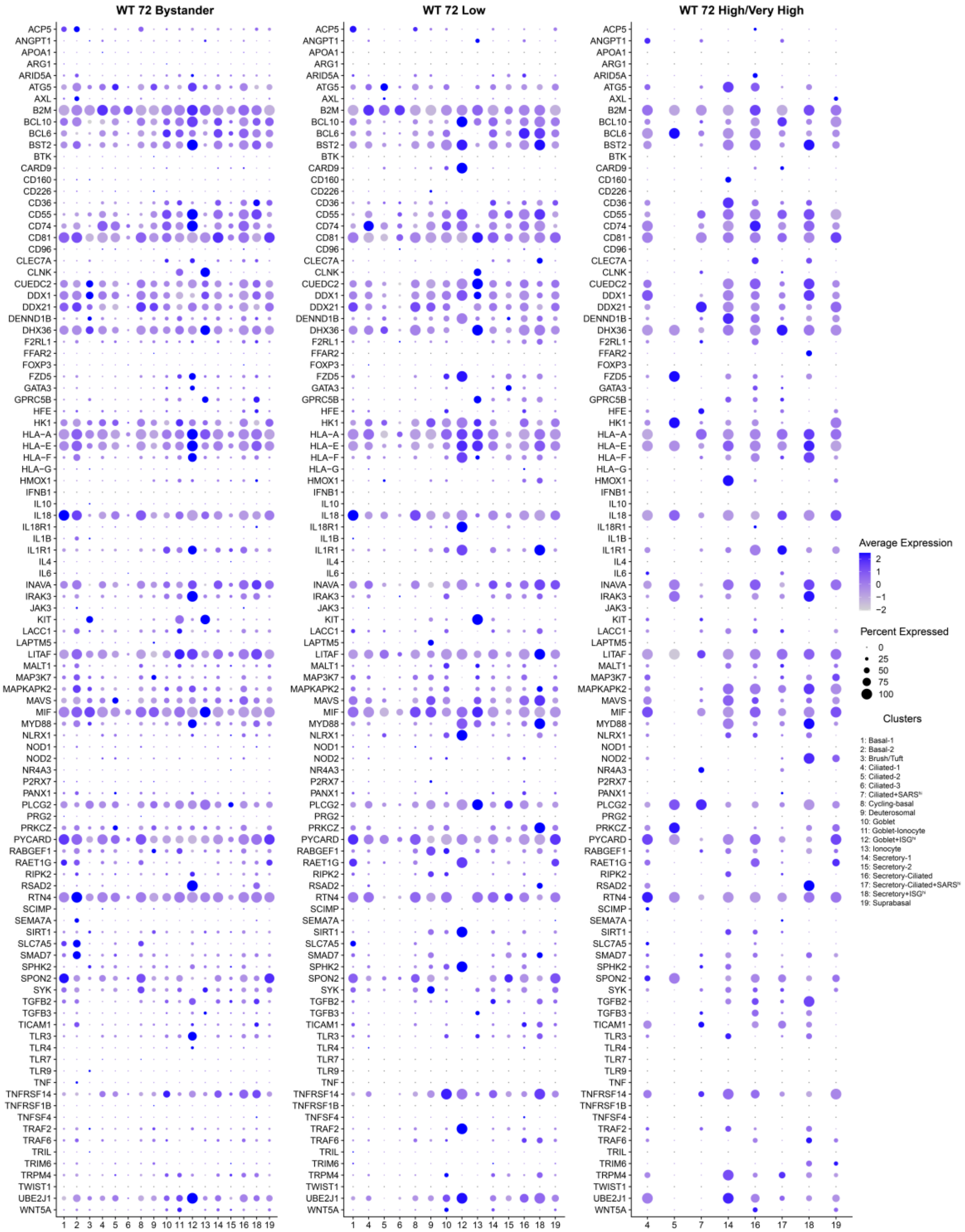

Figure S8. Dot plot showing average expression of cytokine-related genes involved in GO biological term (*cytokine production involved in immune response*) separated into bystander, low and high/very high infection categories in WT 72 hpi adult datasets, related to Figures 3a-b. X-axis shows the number representative of each cell-type cluster (shown in legend) and Y-axis shows the genes involved in the GO biological term (*cytokine production involved in immune response*). The color of the bubble represents the average expression level, and the size of the bubble represents the percentage of cells in each cluster expressing the gene.

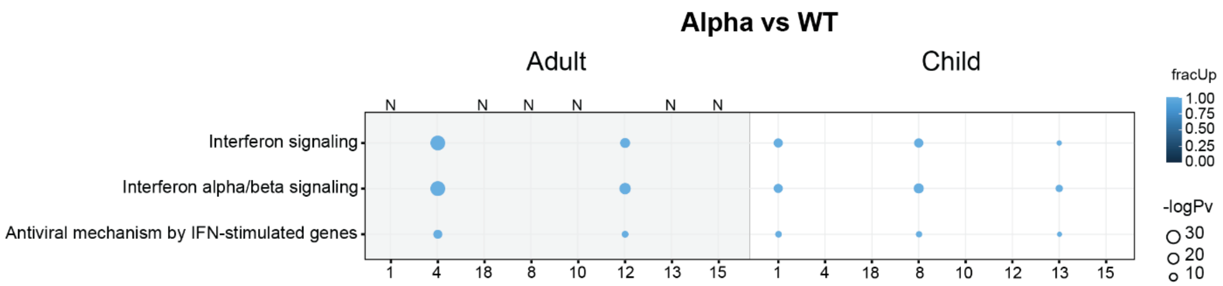

Figure S9. Significantly enriched reactome pathways analyzed using *multiGO* using significant DE results from comparing Alpha- vs WT-infected ALI-cultures, related to Figure 5. Columns with no matching DE data available are denoted with 'N'. Bubble size indicates -log10 enrichment p-values, and the color of the bubble indicates the proportion of upregulated genes involved in the pathway (i.e. fracUp). A subset of the results from the top 35 terms are shown. Same numbering of cell clusters shown in the legend in **Figure S6** are used. Thresholds of pv_thresh=0.05, enrichment pv_thresh=0.005 and logFC_thresh=1 were used. A full list of enriched reactome pathways is available via the link in **Table S3**.

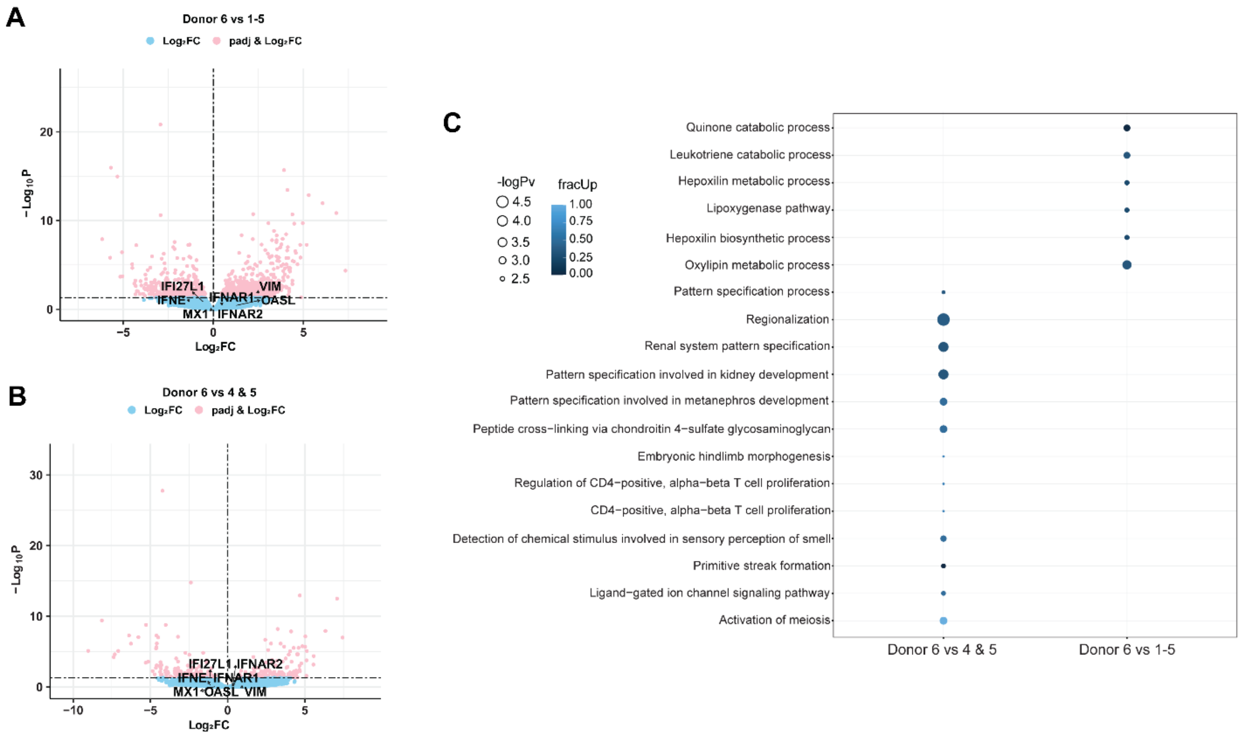

Figure S10. Expression of *VIM* and immune profiles within mock-control cells across donors, related to STAR Methods. (A-B) DE genes comparing mock-control cells from donor 6 vs **A)** all other donors and **B)** other child donors. *VIM* is upregulated in donor 6 vs donors 1-5, but not compared with only the child donors. X-axis shows the $\log_2$FC and Y-axis shows the $-\log_{10}p_{adj}$, with cut-offs at $p_{adj}=0.05$. Dots in blue show the genes which did not meet the threshold of $p_{adj} = 0.05$, and dots in pink show the genes which met the threshold. **C)** *MultiGO* output of enriched GO biological terms in mock-control donor 6 against all other donors and against other child donors. Thresholds of $p_{adj} < 0.05$, enrichment p-value < 0.005 and |log2FC| > 1 were used. Bubble size indicates $-\log_{10}$ enrichment p-values, and the color of the bubble indicates the proportion of upregulated genes involved in the term (i.e. fracUp).

Table S1. Characteristics of each cell-type with multiple sub-clusters (incl. GO biological terms), related to Figure 2a. Determined by *ShinyGO* with DE genes determined by the *Seurat* 'FindAllMarkers' function (**STAR Methods**).

Cell-type	Characteristics
**Secretory-1**	Ethanol oxidation<br>Retinoic acid metabolic proc.<br>Reg. of neural precursor cell proliferation
**Secretory-2**	**Decreased:**<br>Ethanol oxidation<br>Detoxification of copper ion<br>Retinoic acid metabolic proc.
**Secretory-3/Secretory+ISG^{hi}**	Neg. reg. of viral genome replication<br>Type I interferon signaling pathway<br>Cellular response to type I interferon
**Basal-2**	High *KRT14*;
**Ciliated-1**	CC phase: G1> G2M/S<br><br>Epithelial cilium movement involved in extracellular fluid movement<br>Extracellular transport<br>Axoneme assembly
**Ciliated-2**	G2M/S > G1 phase (ciliated) - PROLIFERATING<br>Reg. of cilium beat frequency<br>Epithelial cilium movement involved in extracellular fluid movement<br>Extracellular transport
**Ciliated-3**	G2M/S > G1 phase (low mitochondrial content) (ciliated) – PROLIFERATING<br>Mitochondrial ATP synthesis coupled proton transport<br>Purine ribonucleoside triphosphate metabolic proc.<br>Oxidative phosphorylation

Table S2. Number of cells involved in each infection tier and percentage of high-very highly infected cells per cell-type, related to Figures 2a & c-e.

Cell-Type	Uninfected	Low	Medium	High	Very High	Total	Medium-Very High	High (%)	Very High (%)	High-Very High (%)
**Suprabasal**	14357	6928	150	75	15	21525	240	0.3	0.1	0.4
**Secretory-1**	13159	3857	452	110	31	17609	593	0.6	0.2	0.8
**Goblet**	5210	710	64	26	11	6021	101	0.4	0.2	0.6
**Goblet-Ionocyte**	56	20	0	1	0	77	1	1.3	0	1.3
**Basal-1**	8983	5149	78	16	5	14231	99	0.1	0	0.1
**Ciliated-1**	10923	2741	262	124	74	14124	460	0.9	0.5	1.4
**Secretory-2**	7521	1281	41	47	6	8896	94	0.5	0.1	0.6
**Ciliated-3**	10970	1576	107	36	2	12691	145	0.3	0	0.3
**Cycling-basal**	6273	2479	49	14	6	8821	69	0.2	0.1	0.2
**Ciliated-2**	5037	1167	62	60	7	6333	129	0.9	0.1	1.1
**Basal-2**	1502	1529	21	8	2	3062	31	0.3	0.1	0.3
**Ciliated+SARS^{hi}**	0	7	21	926	981	1935	1928	47.9	50.7	98.6
**Secretory-Ciliated+SARS^{hi}**	2	9	57	392	245	705	694	55.6	34.8	90.4
**Goblet+ISG^{hi}**	53	488	56	17	4	618	77	2.8	0.6	3.4
**Secretory+ISG^{hi}**	126	1145	100	41	25	1437	166	2.9	1.7	4.6
**Deuterosomal**	1458	344	33	27	17	1879	77	1.4	0.9	2.3
**Ionocyte**	803	330	12	4	4	1153	20	0.3	0.3	0.7
**Brush/Tuft**	453	108	0	1	0	562	1	0.2	0	0.2
**Secretory-Ciliated**	1092	229	47	45	36	1449	128	3.1	2.5	5.6

Table S3. ***multiGO*** **links to full results of GO biology terms/reactome pathway enrichment analysis for DGE results, related to Figures 3-5.**

multiGO analysis	Type	Link
**Alpha vs WT**	GO	https://coinlab.mdhs.unimelb.edu.au/multigo3/?dir=multiGO_sc/recluster_Jan_23_edgeR_LRT_2/Alpha_vs_WT.zip&go_type=biological_process&reorder=FALSE&go_thresh=0.005&pvthresh=0.05&reorder=FALSE&fc_thresh=1&max_go=75
**Alpha vs WT**	Reactome	https://coinlab.mdhs.unimelb.edu.au/multigo3/?dir=multiGO_sc/recluster_Jan_23_edgeR_LRT_2/Alpha_vs_WT.zip&go_type=ReactomePathways&reorder=FALSE&go_thresh=0.005&pvthresh=0.05&reorder=FALSE&fc_thresh=1&max_go=35
**Inf vs bystander**	GO	https://coinlab.mdhs.unimelb.edu.au/multigo3/?dir=multiGO_sc/recluster_Jan_23_edgeR_LRT_2/inf_vs_bystander.zip&go_type=biological_process&reorder=FALSE&go_thresh=0.005&pvthresh=0.05&reorder=FALSE&fc_thresh=1&max_go=35
**Inf vs bystander**	Reactome	https://coinlab.mdhs.unimelb.edu.au/multigo3/?dir=multiGO_sc/recluster_Jan_23_edgeR_LRT_2/inf_vs_bystander.zip&go_type=ReactomePathways&reorder=FALSE&go_thresh=0.005&pvthresh=0.05&reorder=FALSE&fc_thresh=1&max_go=100
**Bystander vs control**	GO	https://coinlab.mdhs.unimelb.edu.au/multigo3/?dir=multiGO_sc/recluster_Jan_23_edgeR_LRT_2/bystander_vs_control.zip&go_type=biological_process&reorder=FALSE&go_thresh=0.005&pvthresh=0.05&reorder=FALSE&fc_thresh=1&max_go=35
**Bystander vs control**	Reactome	https://coinlab.mdhs.unimelb.edu.au/multigo3/?dir=multiGO_sc/recluster_Jan_23_edgeR_LRT_2/bystander_vs_control.zip&go_type=ReactomePathways&reorder=FALSE&go_thresh=0.005&pvthresh=0.05&reorder=FALSE&fc_thresh=1&max_go=35
**Inf vs uninf**	GO	https://coinlab.mdhs.unimelb.edu.au/multigo3/?dir=multiGO_sc/recluster_Jan_23_edgeR_LRT_2/inf_vs_uninf.zip&go_type=biological_process&reorder=FALSE&go_thresh=0.005&pvthresh=0.05&reorder=FALSE&fc_thresh=1&max_go=35
**Inf vs uninf**	Reactome	https://coinlab.mdhs.unimelb.edu.au/multigo3/?dir=multiGO_sc/recluster_Jan_23_edgeR_LRT_2/inf_vs_uninf.zip&go_type=ReactomePathways&reorder=FALSE&go_thresh=0.005&pvthresh=0.05&reorder=FALSE&fc_thresh=1&max_go=35
**Immune profiles/*VIM***	GO	https://coinlab.mdhs.unimelb.edu.au/multigo3/?dir=multiGO_sc/recluster_JAN_23_limma/immune_profiles_mixed_model_donor.zip&go_type=biological_process&reorder=FALSE&go_thresh=0.005&pvthresh=0.05&reorder=FALSE&fc_thresh=1&max_go=35

Table S4. List of potential amino acid changes between GISAID references and viral stock utilized in this study, related to STAR Methods.

Stock strain	Genomic mutations compared with GISAID	Additional amino acid sequence changes compared with GISAID	Subtractive amino acid sequence changes compared with GISAID
VIC01	C--> T position 23525	S:H655Y	N/A
VIC17991	AGGG --> AGG position 4206, C --> T position 11687, GCCC --> GCC position 29731	ORF1a:G1333-	ORF1a:A1708D, ORF1a:I2230T, ORF1a:S3675-, ORF1a:G3676-, ORF1a:F3677-, S:H69-, S:V70-
